# Differential Modulation of Functional Dynamics and Allosteric Interactions in the Hsp90-Cochaperone Complexes with p23 and Aha1: A Computational Study

**DOI:** 10.1371/journal.pone.0071936

**Published:** 2013-08-19

**Authors:** Kristin Blacklock, Gennady M. Verkhivker

**Affiliations:** 1 School of Computational Sciences and Crean School of Health and Life Sciences, Schmid College of Science and Technology, Chapman University, Orange, California, United States of America; 2 Department of Pharmacology, University of California San Diego, La Jolla, California, United States of America; Weizmann Institute of Science, Israel

## Abstract

Allosteric interactions of the molecular chaperone Hsp90 with a large cohort of cochaperones and client proteins allow for molecular communication and event coupling in signal transduction networks. The integration of cochaperones into the Hsp90 system is driven by the regulatory mechanisms that modulate the progression of the ATPase cycle and control the recruitment of the Hsp90 clientele. In this work, we report the results of computational modeling of allosteric regulation in the Hsp90 complexes with the cochaperones p23 and Aha1. By integrating protein docking, biophysical simulations, modeling of allosteric communications, protein structure network analysis and the energy landscape theory we have investigated dynamics and stability of the Hsp90-p23 and Hsp90-Aha1 interactions in direct comparison with the extensive body of structural and functional experiments. The results have revealed that functional dynamics and allosteric interactions of Hsp90 can be selectively modulated by these cochaperones via specific targeting of the regulatory hinge regions that could restrict collective motions and stabilize specific chaperone conformations. The protein structure network parameters have quantified the effects of cochaperones on conformational stability of the Hsp90 complexes and identified dynamically stable communities of residues that can contribute to the strengthening of allosteric interactions. According to our results, p23-mediated changes in the Hsp90 interactions may provide “molecular brakes” that could slow down an efficient transmission of the inter-domain allosteric signals, consistent with the functional role of p23 in partially inhibiting the ATPase cycle. Unlike p23, Aha1-mediated acceleration of the Hsp90-ATPase cycle may be achieved via modulation of the equilibrium motions that facilitate allosteric changes favoring a closed dimerized form of Hsp90. The results of our study have shown that Aha1 and p23 can modulate the Hsp90-ATPase activity and direct the chaperone cycle by exerting the precise control over structural stability, global movements and allosteric communications in Hsp90.

## Introduction

**Figure 1:**
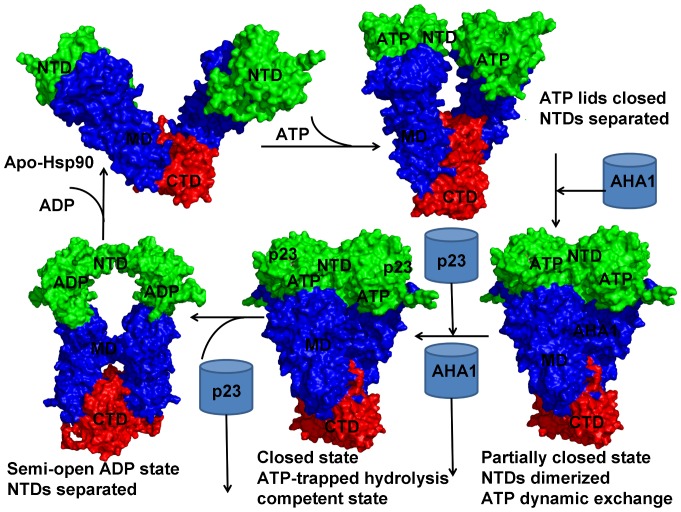
The Functional Role of the Cochaperones Aha1 and p23 in the Hsp90-ATPase Cycle. Clockwise from top left, ATP binding to the Hsp90-N domain of apo-HSP90 induces a conformational change and the closure of the ATP lid leading to a “semi-closed” state with twisted subunits and partly separated Hsp90-N domains. This state is illustrated by using a twisted conformation of the mammalian Grp94 homologue from the crystal structure complex with AMP-PNP (PDB ID 2O1U) [40]. The unbound form of the chaperone is depicted by using the crystal structure of the bacterial homologue HtpG in an apo-form (PDB ID 2IOQ) [39]. After lid closure, Aha1 accelerates the ATPase cycle facilitating dimerization process of the Hsp90-N domains and the formation of a partially closed state with the dynamically associated ATP. Binding of p23 displaces Aha1 and stabilizes the completely closed ATP-bound Hsp90 dimer in a hydrolysis-competent state. This conformation is committed for ATP hydrolysis. Both states are represented by the closed dimer conformation from the crystal structure of yeast Hsp90 bound to the AMP-PNP and p23 (mammals)/Sba1 (yeast homologue) [38]. After ATP hydrolysis, p23 is released leading to a “semi-opened” ADP-bound state with the Hsp90-N domains partially separated. This functional state is presented by the crystal structure of an ADP-bound form of the bacterial homologue HtpG (PDB ID 2IOP) [39]. After ADP release, the substrate clients are released and the Hsp90-N domains dissociate leading to an open free form of the chaperone (pdb id 2IOQ). The Hsp90 structures are shown in surface representation and colored according to their domain nomenclature : N-terminal ATPase domain (Hsp90-N) that binds ATP is shown in green; a middle domain (Hsp90-M) that binds cochaperones and client proteins is shown in blue, and a C-terminal domain (Hsp90-C) required for dimerization is shown in red.

Molecular chaperones are essential proteins that have evolved to assist and facilitate conformational development, folding and stability of a diverse repertoire of proteins inside the cell [1]–[7]. The molecular chaperone Hsp90 (90 kDa heat-shock protein) is highly conserved in a variety of organisms and plays a central role in folding, assembly, and quality control of many client proteins, including but not limited to hormone receptors, transcription factors, and protein kinases. The Hsp90 chaperone machinery is supported by a large cohort of cochaperones that act cooperatively in protecting activation-competent protein states from degradation and aggregation [8]–[13]. The regulatory complexes of Hsp90 with cochaperones have also evolved to regulate diverse functions of chaperone clients by facilitating their evolutionary development and sustaining the pressure of detrimental mutations that otherwise might lead to the production of unstable or inactive proteins [14]–[16]. The integration of cochaperones into the Hsp90 system is driven by the regulatory mechanisms that modulate the ATPase activity and control coupling of the Hsp90-ATPase cycling machine to the loading, activation, and release of client proteins [8]–[16]. The proposed functional classification of Hsp90 cochaperones into client recruiters (Sti1/Hop, Cdc37, Sgt1), remodelers of Hsp90 (Aha1, FKBP51/FKBP52), and late-acting cochaperones (p23) has highlighted distinctive functional roles of cochaperones in supporting Hsp90 activities [17]. The recent structural and functional investigations have revealed a diversity of mechanisms by which cochaperones can intervene in the progression of the Hsp90-ATPase driven changes, often by directing the chaperone cycle towards specific intermediate states (Figure 1). These cochaperones can often have overlapping functions by regulating the rate of ATP hydrolysis (Aha1, Cdc37, p23), modulating conformational flexibility of Hsp90 (p23, Sgt1) and recruiting specific protein clients to the Hsp90 system (Sti1/Hop, Cdc37, Sgt1) [18]–[30]. Aha1 (Activator of Heat shock 90 kDa protein ATPase homolog 1) is a cochaperone which stimulates the ATPase activity [17], [18].

A number of cochaperones such as p23/Sba1p [19]–[23] Sti1p/Hop [24]–[26], and Cdc37 [29], [30] can arrest the Hsp90-ATPase cycle in a particular conformational state to recruit and support activities of specific clients. The late-acting cochaperone p23 can slow down the rate of ATP hydrolysis by binding selectively to the ATP-bound closed form of Hsp90 and “delaying” the release of client proteins undergoing maturation [19]–[23]. The client recruiter cochaperone Cdc37 functions as a highly specialized adaptor that can deliver kinase clients to the chaperone system and, in coordination with Hsp90, can promote and maintain stabilization of protein kinases during the maturation process [29], [30]. Allosteric regulation of protein clients by the Hsp90 machine underlies its fundamental role in signal transduction networks associated with protein synthesis, assembly and activation of key signaling proteins driving tumor development and progression [31]–[34]. Deregulation of oncogenic pathways is often linked to a plethora of protein kinase clients regulated by the Hsp90-Cdc37 system. Allosteric inhibition of the Hsp90 machinery may lead to a combinatorial blockade of multiple oncogenic nodes holding a promise as the emerging therapeutic strategy against multiple kinase-dependent cancers. [35]–[37].

**Figure 2:**
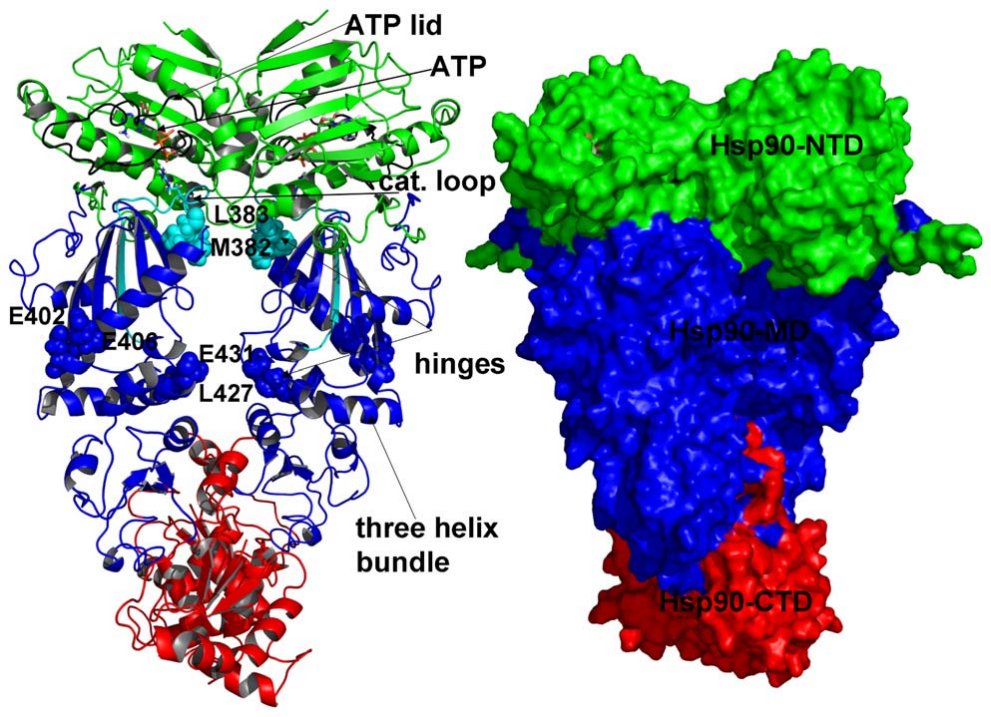
The Structure of the Full-Length Hsp90 Dimer. The homodimer architecture of the full-length Hsp9p0 dimer is illustrated by the crystal structure of a closed ATP-bound conformation of yeast Hsp90 dimer [38]. The structure is shown in a ribbon representation with a detailed annotation of structural elements (left panel) and in a general surface-based protein representation (right panel). The Hsp90-N domain is shown in green; the Hsp90-M domain is depicted in blue and the Hsp90-C domain is presented in red. Left Panel: The ATP lid residues 95–123 are shown in black (95-TIAKSGTKAFMEALSAGADVSMIGQFGVG-123). The catalytic loop residues (371-SEDLPLNLSREMLQQ-385) are shown in cyan with a key catalytic residue R-380 highlighted in cyan sticks. ATP molecule is colored by atoms and is shown in sticks. The three-helix bundle shown in blue ribbons (helix 1: residues 386–408; helix 2: residues 412–431; helix3: residues 435–442) links the inter-domain regions and the regulatory hinges. The N-M inter-domain hinge residues M382 and L383 are shown in cyan spheres; the M-C inter-domain hinge residues E402 and E406 are shown blue spheres. The cyan color of M382/L383 is according to the coloration of the catalytic loop (residues 371–385). The blue color of E402/E406 is according to the coloration of the Hsp90-M domain. In the original crystal structure of yeast Hsp90 the domain annotation for the monomer 1 is: Hsp90-N (residues 2–216), Hsp90-M (residues 262–329, 339–526) and Hsp90-C (residues 527–597, 611–677). In the monomer 2 Hsp90-N (residues 2–216), Hsp90-M (residues 262–526) and Hsp90-C (527–597, 611–677). The structurally unresolved residues are 217–261, 330–338 (only in the first monomer), and 598–610. The Pymol program was used for visualization of Hsp90 structures (The PyMOL Molecular Graphics System, Version 1.2r3pre, Schrödinger, and LLC).

The crystal structures of a Hsp90-cochaperone complex from yeast [38], E. coli HtpG [39], a Grp94 homologue-nucleotide complex [40] and an Hsp90-client protein complex [41] have revealed a homodimer chaperone architecture with three well-defined domains: a highly conserved N-terminal ATPase domain (Hsp90-N) responsible for ATP binding, a middle domain (Hsp90-M), which competes for client binding, and a C-terminal domain (Hsp90-C) required for dimerization (Figure 2). Combined with biophysical approaches, such as hydrogen exchange mass spectrometry (HX-MS), single-molecule fluorescence resonance energy transfer (FRET), electron microscopy, and small-angle X-ray scattering (SAXS), these studies have characterized conformational changes in Hsp90 during the ATPase cycle [42]–[49]. Among dynamic “hallmarks” of Hsp90 is the existence of highly coordinated motions of different domains that regulate opening-closing transitions of Hsp90, most notably the emerging evidence of anti-correlated motions between a C-terminal open and an N-terminal closed states [44].

These studies have demonstrated that the Hsp90 dimer “lives” in an ATP-driven functional cycle operating by a stochastic switching mechanism based on spontaneous fluctuations between structurally different functional states [50]–[52]. In the course of the cycle, ATP binding leads to an N-terminal dimerized form of Hsp90 and the formation of the “molecular clamp”, followed by subsequent hydrolysis and dissociation of the Hsp90-N domains (Figure 1). The rate-limiting step that drives kinetics of these conformational changes is the formation of the closed N-terminal dimerized state rather than ATP hydrolysis [53]. Whereas the ATPase cycle of the bacterial homologue HtpG is strongly coupled to the nucleotide turnover and driven by a mechanical ratchet mechanism, the rate-limiting N-terminal conformational changes in yeast Hsp90 are nucleotide-independent and determined by slow thermal fluctuations between the open and closed states [54]. Moreover, only binding of cochaperones may turn the stochastically-based conformational changes into an orderly and successive progression of the ATPase cycle for yeast Hsp90 [53], [54]. The synergistic action of the cochaperones Aha1 and p23 is crucial for regulation and progression of the Hsp90 ATPase cycle (Figure 1). Aha1 accelerates the ATPase cycle by biasing Hsp90 towards an N-terminally closed ATP-bound conformation immediately preceding ATP hydrolysis [17], [18]. Binding of p23 displaces Aha1 and stabilizes the completely closed ATP-trapped Hsp90 dimer in a hydrolysis-competent state [19]–[23]. After ATP hydrolysis, p23 and the activated client are released (Figure 1). In the absence of p23, Aha1-induced stimulation of the ATPase cycle would lead to accelerated ATP hydrolysis [17]. Hence, Aha1 and p23 can modulate the thermodynamic and kinetic properties of the ATPase cycle to achieve temporal control over conformational transitions required for proper loading and release of the substrates from the Hsp90 system (Figure 1).

The first X-ray crystal structure of the full-length Hsp90 dimer was obtained from the co-crystal structure of yeast Hsp90 bound to the AMP-PNP and two molecules of p23 (mammals)/Sba1 (yeast homologue) [38]. The dynamics of the human Hsp90-p23 complex in solution was studied by combination of NMR spectroscopy and native mass spectrometry [55]–[57]. These experiments have revealed that the chaperone constructs that contained both Hsp90-N and Hsp90-M domains were capable of binding to p23, whereas the Hsp90-N domains alone could not form high affinity complexes with p23 even in the presence of ATP [55], [56]. Collectively, the results of crystallographic and NMR investigations have concluded that (a) p23 binding can induce significant long-range conformational changes in the Hsp90-M domains; (b) the ATP-driven dimerization can be allosterically supported by the p23-induced stabilization of the Hsp90-N and Hsp90-M domains.

Unlike p23, Aha1 can accelerate the progression of the Hsp90-ATPase cycle towards a dimerized closed form by directly “dialing” into the equilibrium motions of Hsp90 and promoting structural rearrangements that favor a closed chaperone form irrespective of the presence of nucleotide [58]–[60]. The initial biochemical studies have characterized Aha1-mediated stimulation of the ATPase activity as primarily determined by the interactions between N-terminal domain of Aha1 (Aha1-N) and the Hsp90-M [58]–[61]. Importantly, the Aha1-N domain appeared to be essential and contributed ∼ 30% of the ATPase activity stimulated by the full-length cochaperone Aha1 [58]. Although the maximal activation can be achieved only by the complete Aha1 construct, the C-terminal domain of Aha1 (Aha1-C) alone does not bind to Hsp90 nor can it stimulate the ATPase activity [58]. Gel filtration chromatography and in vitro assays of yeast Aha1 binding to yeast Hsp90 have indicated that Aha1 binds to the Hsp90-M and stimulates the ATPase activity of the molecular chaperone [59]. Structure-functional analysis of yeast Hsp90 using co-precipitation and isothermal titration calorimetry have confirmed that the primary docking site of Aha1 is proximal to the catalytic loop in the Hsp90-M [61]. These studies have suggested that Aha1 may allosterically enhance the interactions of the Hsp90-M and the Hsp90-N domains and thereby facilitate conformational changes towards the closed form of Hsp90. The recent functional studies have also revealed that Aha1 may form complexes with Cdc37 and Hsp90 in the absence of nucleotides [62], [63]. However, Aha1 binding can dramatically reduce Cdc37 affinity to Hsp90 and the addition of ATP analogs could trigger a full displacement of Cdc37 from the chaperone complex [64], [65].

The crystal structures of the yeast Aha1-N (residues 1–153) in the unbound form and in the complex with the yeast Hsp90-M domain (residues 273–530) [66], [67] have revealed that Aha1-N binding can release the catalytic loop residues from the intra-molecular interactions and bring them closer to the nucleotide site by adopting a partially open active orientation [66]. Structural details of the Hsp90-Aha1 interactions have been elucidated for the full length Hsp90 in a series of NMR and FRET experiments that have revealed an asymmetric binding of a single Aha1 molecule with the Hsp90 dimer sufficient for stimulation of the ATPase activity [68]. These studies have confirmed the primary contacts between the Aha1-N and the Hsp90-M domains, which are nucleotide-independent, whereas binding of the Aha1-C with the Hsp90-N can occur only in the presence of ATP. Structural and dynamic basis of Aha1 regulation for the full-length mammalian Hsp90 and Aha1 was also investigated using cross-linking and mass spectrometry biophysical analyses [69]. The obtained molecular footprint of the Hsp90-Aha1 interactions has suggested that Aha1 may fluctuate between open and closed states of Hsp90 during the initial recognition event, while the thermodynamic bias would force Aha1 to be trapped in a partially or fully closed Hsp90 dimer [69]. Collectively, these studies have demonstrated that Aha1-mediated acceleration of the Hsp90-ATPase activity requires the complete Aha1 for full activation and may operate via a sequential binding mechanism of the Aha1-N and Aha1- C domains to Hsp90 [68], [69].

The most recent analysis of cochaperone integration during the Hsp90 ATPase cycle was addressed by using a combination of biochemical, biophysical and *in vivo* methods [70]. This study has revised a general stochastic mechanism of the ATPase cycle by recognizing that the integration of cycle-accelerating cochaperones (Aha1, Cpr6) and cycle-inhibiting cochaperones (p23) could impose certain directionality into the Hsp90 cycle to ensure a steady progression between intermediates towards the active closed dimer required for the maturation of client proteins. A global fingerprint of the Hsp90 regulated proteome has been recently explored by global proteome profiling [71]–[73] and cell-based interrogation exploiting proteomic response to Hsp90 inhibitors [74]–[76]. However, despite technological advances in probing molecular mechanisms of the Hsp90 regulation, a dynamic nature of the Hsp90 interactions with cochaperones and client proteins remains elusive, making structural and energetic characterization of the Hsp90 mechanisms challenging even for modern experimental techniques. These difficulties are compounded by recent findings that molecular and energetic determinants of the Hsp90 interactions are not only complex but are also broadly distributed in structural space [73]. Frustrated by the diversity and transience of the Hsp90 interactions, prior studies have left a number of gaps in understanding how cochaperones can modulate allosteric signaling and regulate activities of the interacting partners.

Structural plasticity and functional adaptation of the Hsp90 chaperone in signaling networks are regulated by allosteric interactions that allow for recognition of a wide range of cochaperones and client proteins with different sequences and conformations. Rapid and efficient transmission of long-range conformational changes plays a vital role in allosteric regulation and may present a common functional requirement encoded across single protein domains and macromolecular assemblies [77]–[83]. Coarse-grained approaches and elastic network models such as Gaussian network model (GNM) [84]–[86] and the anisotropic network model (ANM) [87] combined with the normal mode analysis (NMA) [88], [89] are indispensable tools that can probe allosteric interactions and functional motions that are primarily determined by the equilibrium structure and could be adequately described by the low frequency normal modes [90]–[92]. A combination of molecular dynamics (MD) simulations and the protein structure network (PSN) analysis using graph-based approaches [93]–[100] can often identify functionally important structural changes in conformational populations of states. Mapping of the dynamical profiles obtained from GNM and NMA approaches with the protein network parameters) [95]–[98] can provide the distribution and reorganization of structurally stable residue networks upon ligand or protein binding. Computational studies have employed these dynamic approaches to model molecular mechanisms of allosteric signaling in the molecular chaperones [101]–[109]. We have recently reported a series of computational investigations revealing the atomic details of the allosteric pathways which may regulate the conformational equilibrium of the Hsp90 chaperone [101]–[107]. These studies have identified conserved functional motifs that act as central regulators of the Hsp90 activity, including control of ATP hydrolysis, the inter-domain communications and protein client binding (Figure 2) [107]. The recognition sites are often positioned close to these regulatory motifs containing catalytic and hinge residues. Based on these findings, we have proposed that functional coupling of structurally stable regulatory motifs and conformationally mobile elements may regulate allosteric binding of cochaperones and protein clients [107]. A similar analysis based on atomistic simulations of the Hsp90 crystal structures from different species has detected two inter-domain hinge sites regulating allosteric interactions of Hsp90 [108]. Force-distribution analysis has identified an internal signaling pathway connecting the nucleotide binding site via a dynamic hinge with the distantly located client binding region in the middle domain of HtpG [109].

Determining the mechanisms by which structural changes elicited by various cochaperones could concertedly lead to the formation of a catalytically active form is of fundamental importance in understanding the activation of chaperone clients. In this work, we report the results of computational modeling of allosteric regulation in the Hsp90 complexes with the cochaperones p23 and Aha1. Integration of protein docking, coarse-grained simulations, protein structure network analysis and the energy landscape modeling has provided a convenient platform for quantifying the regulatory principles underlying cochaperone modulation of the Hsp90 activity. Although the network of allosteric communications is determined by the structural architecture of Hsp90, we show that functional motions of Hsp90 can be selectively modulated by cochaperones via specific targeting of the regulatory hinges that could stabilize specific chaperone conformations. Using protein structure network analysis, we characterize cochaperone-induced reorganization of structurally stable networks that could allow p23 and Aha1 to readily alter the conformational equilibrium of Hsp90 and stabilize specific functional states. The results of this study shed some light on how structural and dynamic changes induced by cochaperones could direct the Hsp90 cycle by progressively stabilizing a catalytically competent dimer required for maturation of client proteins.

## Results/Discussion

According to the central hypothesis of this study allosteric regulation of the diverse chaperone functions is encoded in the topology of the Hsp90 fold, but can be selectively modulated and activated by cochaperones. In this model, allosteric interactions provide a mechanism for coordinated collective motions and structural adaptation of Hsp90 to binding partners via equilibrium switching to the preferential conformational state. We extended and tested this mechanistic model in a detailed computational investigation of Hsp90 binding with the p23 and Aha1 cochaperones. The following specific objectives and research strategies were pursued in the present work: (a) using data-driven protein docking approach and MD refinement we assembled structures of the Hsp90 complexes and reconstructed binding interfaces of Hsp90 with p23 and Aha1; (b) the predicted models of the Hsp90 complexes were then used by the GNM and NMA approaches to characterize collective motions and normal modes of the chaperone and assess the differential impact of the individual domains and multi-domain constructs of p23 and Aha1 on functional dynamics of Hsp90; (c) we analyzed how functional motions of Hsp90 determined by the physical constraints of the homodimer fold could be modulated by p23 and Aha1; (d) we characterized the distribution and reorganization of structurally stable interaction networks in the Hsp90-cochaperone complexes using dynamic network parameters (clusters, hubs, cliques and communities) averaged over the MD trajectories; (e) we also carried out the energy landscape analysis of the Hsp90-cochaperone complexes and demonstrated that targeted modulation of the local frustration profiles in complexes with Aha1 could serve as an energetic detector of hotspot residues critical in allostetric regulation.

### Structure and Dynamics of the Hsp90-p23 Interactions: Allosteric Stabilization of the Hsp90 Dimerized State

**Figure 3:**
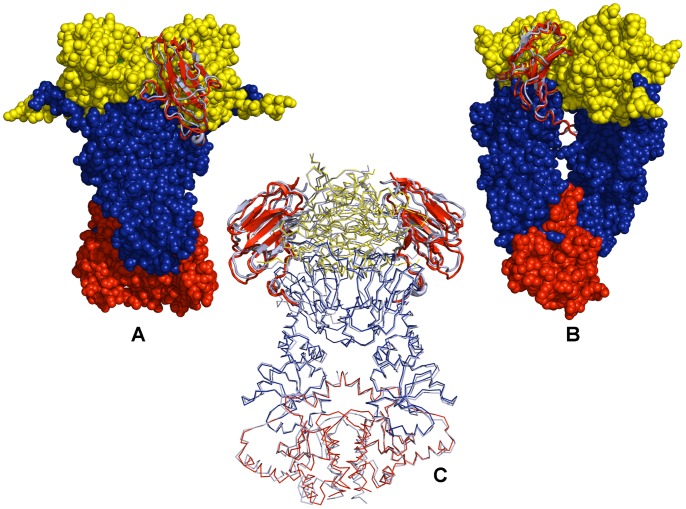
Structural Modeling of the Hsp90-p23 Complex. Three-body body docking simulations used as independent unbound molecules the ATP-bound form of the Hsp90 dimer and the crystallographic conformations of two p23 molecules. Superposition of the docked Hsp90-p23 complex and the crystal structure of yeast Hsp90 bound the AMP-PNP and p23. The crystal structure has revealed two cochaperone molecules bound to the closed dimer form of Hsp90. (A, B) Front and side views of the docked Hsp90-p23 complex. The Hsp90 structure (pdb id 2CG9) is shown in spheres; the Hsp90-N domain in green, the Hsp90-M domain in blue, and the Hsp90-C domain in red. To illustrate binding of p23 molecules in a depression area at the interface of the two Hsp90-N domains, the crystallographic conformations of p23 (in red) and the predicted lowest docked conformations (in cyan) are highlighted in ribbons. (C) Superposition of the predicted yeast Hsp90-p23 complex (in blue) with the docked p23 molecules in cyan and the crystal structure (in green) with the crystallographic p23 conformations in red.

We began by discussing the results of structural modeling and functional dynamics analysis of the Hsp90-p23 interactions. The crystal structure of the Hsp90-p23/Sba1p complex has revealed two cochaperone molecules occupying the depression at the interface of the two Hsp90-N domains in the closed dimer form [38]. We used the high ambiguity-driven docking approach HADDOCK [110] that converted this structural information into of Ambiguous Interaction Restraints (AIR) templates (Table S1) to guide docking of protein complexes. To validate this approach, we carried out three-body docking simulations using as independent unbound molecules the ATP-bound form of the Hsp90 dimer and the crystallographic conformations of two p23 molecules. The ensemble of generated solutions was subjected to stages of HADDOCK refinement and clustering (see Materials and methods for more details). Simulations converged to the low-energy docked structure that was within root mean square deviation (RMSD) = 1.85 Å from the crystal structure. Subsequent MD-based refinement of the low-energy docked solutions produced the final conformation of the trajectory residing within RMSD = 1.137Å from the crystal structure of the complex (Figure 3). The average RMSD of the MD refinement trajectory from the crystal structure of the complex was ∼1.485 Å.

The predicted conformation of the Hsp90-p23/Sba1p complex was used to characterize conformational mobility profiles in the unbound and bound forms of Hsp90. These results were compared with the NMR analysis of the human Hsp90β-p23 complex [56], [57]. Of particular interest was functional dynamics profiling in the regions that exhibited chemical shift perturbations as a result of Ile-specific NMR labeling experiments in human Hsp90β [56]. Using structure-sequence alignment of yeast and human Hsp90 [111] and sequence analysis of Hsp90 proteins in various organisms [112], we mapped these p23-mediated changes between human Hsp90β and yeast Hsp90.

**Figure 4:**
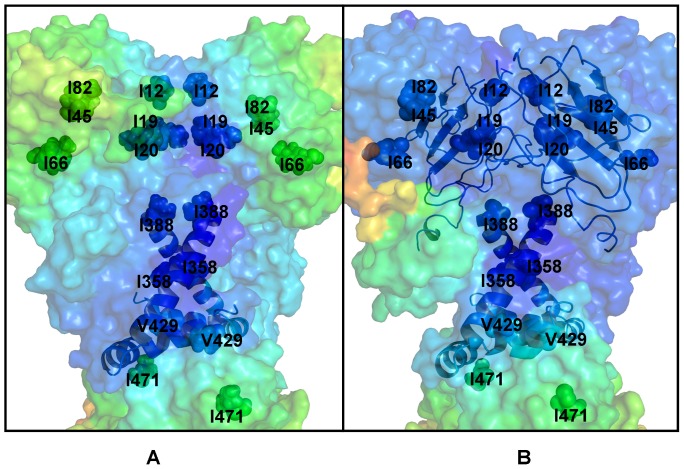
Functional Dynamics and Conformational Mobility Profiles of the ATP-bound Hsp90 Dimer and the Hsp90-p23 Complex. Structural distribution of conformational mobility in the ATP-bound form of yeast Hsp90 (A) and the Hsp90-p23 complex (B) was averaged over three lowest frequency modes obtained from the functional dynamics analysis. A close-up view of the protein mobility profiles for a panel of Ile residues probed in the Ile-targeted NMR experiments of the Hsp90-p23 complex [56]. These residues were mapped onto the crystal structure of yeast Hsp90 (PDB ID 2CG9) and depicted in colored spheres according to their mobility. A surface-based protein representation is employed. The color gradient from blue to red indicates the decreasing structural rigidity (or increasing conformational mobility) of protein residues. The numbering of the Ile-probed residues corresponds to the crystal structure of yeast Hsp90. The highlighted Ile residues in yeast Hsp90 (I12, I19, I20, I45, I66, I82, I358, I388, V429, and I471) correspond to the Ile-probed residues in human Hsp90β (I20, I27, I28, I53, I75, I90, I369, I399, I440, and I482 respectively [56]). The three-helix bundle that links the inter-domain N-M and M-C regions and coordinates motions of the regulatory hinges is shown in ribbon representation. The unrelated obstructing features in the foreground were omitted for clarity. The Pymol program was used for visualization of the protein structures (The PyMOL Molecular Graphics System, Version 1.2r3pre, Schrödinger, and LLC).

We found that binding-induced modifications in the functional dynamics of Hsp90 may affect the global allocation of structurally rigid and conformationally mobile elements (Figures 4). This effect could be seen in the changed structural environment of the Hsp90-N residues, manifested in the increased stabilization of the regions surrounding the NMR-probed Ile residues. According to the NMR data [56], the ATP-dependent changes could be restricted to the Hsp90β-N residues I53, I75, I90 residues from the ATP binding pocket (corresponding to I45, I66, and I82 in yeast Hsp90). While the structural environment of these residues in the ATP-bound dimer consists of structurally rigid and conformationally mobile elements (Figure 4A), this region becomes increasingly rigid and largely homogeneous in the Hsp90-p23 complex (Figure 4B). By monitoring Ile-specific changes in conformational mobility we also observed allosterically induced structural immobilization of the I12, I19, I20 residues in yeast Hsp90 that belong to the helical regions involved in the N-terminal dimerization (the Hsp90β-N residues I20, I27, and I28 respectively) (Figure 4). In agreement with the NMR results, we found that the allosteric effect of p23 binding could propagate over long range distances and considerably strengthen structural rigidity of the Hsp90-M domains and the N-M inter-domain interactions. The enhanced structural rigidity of the yeast Hsp90-M residues I358, I388, V429, and I471 (corresponding to the NMR-probed Hsp90β-M residues I369, I399, I440, and I482) that are distant from the binding site (Figure 4B) is indicative of long-range allosteric changes mediated by p23 binding [56]. The increased structural stability of the Hsp90-M residues L315, I388 and V391, which are proximal to the catalytic R380, may also enforce the closed state of the catalytic loop and further stabilize the ‘inhibited’ form of Hsp90 that is committed to hydrolyzing ATP. It is therefore possible that the ATP-driven dimerization of the Hsp90-N domains can be allosterically enhanced by p23 binding. Consistent with the crystallographic [38] and NMR data [55]–[57], we determined that p23-mediated changes are primarily manifested in (a) structural immobilization of the Hsp90-N residues near the dimerization regions; (b) long-range allosteric changes propagated to the Hsp90-M regions, and (c) the increased association of the Hsp90-N and Hsp90-M domains (Figure 4).

We also examined the distribution of structural rigid and flexible elements at the inter-domain N-M and M-C boundaries, assuming that localized targeted changes in these regions may be responsible for modulation of the ATPase activity. A number of functionally important residues, including the NMR-probed I388 and V429 residues in the Hsp90-M domain, belong to the three-helix bundle (helix 1: residues 386–408; helix 2 : residues 412–431; helix3: residues 435–442) that links the inter-domain regions and the regulatory hinges. The first primary hinge (residues 374-LPLNLSREML-383) is located at the N-M interface (Figure 2) and this region becomes more rigid and homogeneous in the Hsp90-p23 complex (Figure 4B). Structural environment of the second inter-domain M-C hinge (residues 426-KLGVHE-431) remained largely similar to the ATP-bound closed dimer (Figure 4). The moderately reduced conformational mobility of Hsp90 near the regulatory hinge sites suggests that p23 would not stifle collective movements but rather slow down the ATPase-driven conformational transitions.

### Modulation of Allosteric Communications by the Cochaperone p23

**Figure 5:**
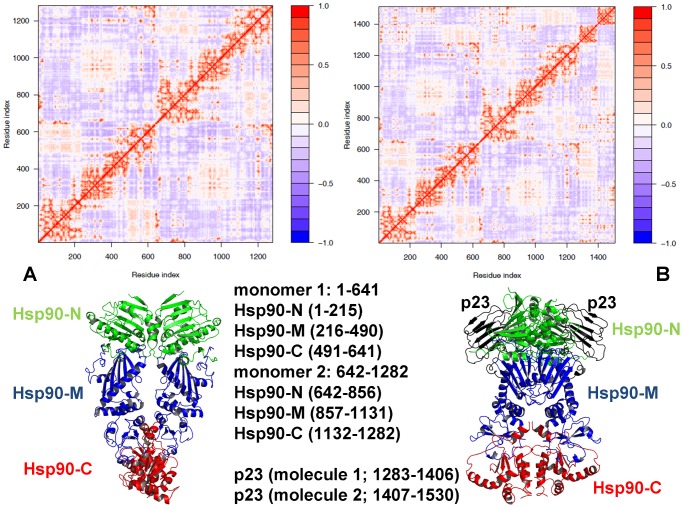
A Comparative Analysis of Correlated Motions in the ATP-bound Hsp90 and the ATP-bound Hsp90-p23 Complex. The cross-correlation matrices of residue fluctuations computed along the low frequency modes in the ATP-bound Hsp90 dimer (A) and in the Hsp90-p23 complex (B). The matrix was calculated using the results of MD-based refinement and NMA of the predicted structures. The essential directions of correlated motions during dynamics were then calculated by diagonalizing the covariance matrix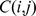. Cross-correlations of residue-based fluctuations vary between +1 (fully correlated motion; fluctuation vectors in the same direction, colored in red) and -1 (fully anti-correlated motions; fluctuation vectors in the same direction, colored in blue). The values above 0.5 are colored in dark red and the lower bound in the color bar indicates the value of the most anti-correlated pairs. Bottom panels: The Hsp90 structure is shown in a ribbon representation (Hsp90-N in green, Hsp90-M in blue, and Hsp90-C in red). The p23 molecules (right bottom panel) are shown in black ribbons. The residue indexing of the Hsp90 domains as used in the cross-correlation matrices is indicated. In the original crystal structure of yeast Hsp90 [38] the domain annotation for the monomer 1 is: Hsp90-N (residues 2–216), Hsp90-M (residues 262–329, 339–526) and Hsp90-C (residues 527–597, 611–677). In the monomer 2 Hsp90-N (residues 2–216), Hsp90-M (residues 262–526) and Hsp90-C (527–597, 611–677). The structurally unresolved residues are 217–261, 330–338 (only in the first monomer), and 598–610. The crystal structure was employed as a starting point for the simulations. The disordered charged loop between the Hsp90-N and Hsp90-M domains was replaced by a modeled Gly-based linker using the ModLoop server [140]. All disordered loops in the Hsp90-M and Hsp90-C domain were modeled with ModLoop by preserving the original protein sequence. The original crystallographic annotation was converted to a consecutive numbering in the cross-correlation matrix where the adjusted domain annotation is as following. The monomer 1 includes residues 1–641 and the monomer 2 consists of residues 642–1282. In the monomer 1: Hsp90-N (residues 1–215), Hsp90-M (residues 216–490) and Hsp90-C (residues 491–641). In the monomer 2: Hsp90-N (residues 642–856), Hsp90-M (residues 857–1131), and Hsp90-C (residues 1132–1282). The p23 residues are 1283–1406 (molecule 1) and 1407–1530 (molecule 2) respectively.

The refined structural models of the Hsp90-p23 complex were analyzed by the elastic network and the NMA approaches. Using these tools we generated the distribution of conformational mobility and the cross-correlation maps of protein residue fluctuations. We compared the cross-correlation matrices of residue fluctuations computed along the low frequency modes in the ATP-bound Hsp90 dimer (Figure 5A) and in the Hsp90-p23 complex (Figure 5B). The slowest modes correspond to the large-scale cooperative movements of the Hsp90 domains, and regions with restricted motions typically serve as focal points for modulating collective domain motions and hinge bending. Of particular interest were the changes in the functional coupling of structurally stable and conformationally flexible residues near the regulatory hinge regions critical for collective motions of Hsp90. The analysis of collective motions was consistent with our earlier studies [102], [107] and recapitulated the global fingerprint of the Hsp90 dynamics that is determined by the chaperone architecture (Figure 5A). Functional motions of the ATP-bound Hsp90 dimer included positive correlations of the Hsp90-N (residues 1–215) and Hsp90-C domains (residues 491–641) within the monomers as well as positive correlations between the Hsp90-M-domains of two monomers. The motions of the Hsp90-C domains were also positively correlated. The evidence of anti-correlated correlated coupling between the Hsp90-N of one monomer (residues 1–215) and the Hsp90-C of the other (residues 1132–1283) (Figure 5A) was consistent with the experimentally observed anti-correlated motions between a C-terminal open and an N-terminal closed state [44]. Overall, the pattern of correlated motions in the Hsp90-p23 complex remained largely intact as compared to the ATP-bound dimer (Figure 5B). A subtle weakening of the intra and inter-monomer couplings, particularly in the positive inter-monomer correlations between the Hsp90-M domains could be still noticed. Interestingly, p23 molecules move concertedly with their bound Hsp90-N domains.

**Figure 6:**
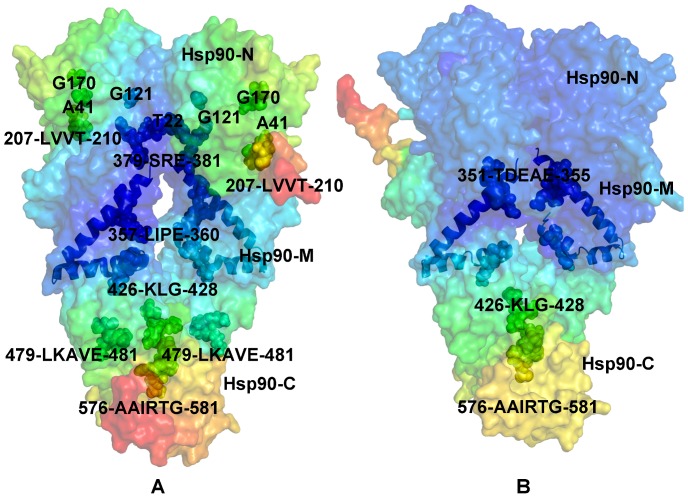
A Comparative Analysis of the Allosterically Communicating Networks. The distributions of allosterically communicating residues are shown in a sphere-based residue representation for the ATP-bound form of Hsp90 (A) and in the Hsp90-p23 complex (B). These distributions are mapped onto respective functional dynamics profiles of Hsp90 shown in a surface-based protein representation. The color gradient from blue to red indicates the decreasing structural rigidity of protein residues similar to Figure 4. The crystal structure of yeast Hsp90 is enveloped in a 50% transparent molecular surface to show the location of the allosterically communicating residues. The location and identity of allosterically communicating residues are annotated and the Hsp90 domains are indicated. The effectively communicating clusters included the Hsp90-N residues (T22, A41, G81, G121, G170, 207-LVVT-210), the Hsp90-M residues (357-LIPE-360, 379-SRE-381, 426-KLG-428, 479-LKAVE-481) and the Hsp90-C residues (576-AAIRTG-581). The changes in the distribution of allosterically interacting residues induced by p23 binding are depicted (B). The close proximity of these residues to the three-helix bundle (shown in ribbon representation) that links the inter-domain regions can be also seen.

These results raise a legitimate question how this seemingly puzzling similarity of collective motions could allow for modulation of the Hsp90-ATPase activity by the p23 cochaperone. To address this, we computed the distributions of allosterically communicating residues in the ATP-bound form of Hsp90 and in the Hsp90-p23 complex that were mapped onto their respective dynamic profiles (Figure 6). In the framework of the GNM approach, the collective motions of the chaperone can also determine communication propensities (CP) of the protein residues, where functionally important sites such as catalytically important residues, active site residues, and regulatory motifs are expected to exhibit rapid and precise communication capabilities [81], [82]. The analysis presented here was based on the GNM approach and directly relates residue fluctuations to their CPs, namely the residues whose distances fluctuate with low intensity communicate with a higher efficiency than the residues with larger fluctuations. In this model, residues with high local interaction density (a high coordination number) are likely to belong to the clusters of effectively communicating residues. While structurally stable residues undergoing cooperative movements may often communicate efficiently, conformationally mobile residues exhibiting a concerted change in their fluctuations may be equally important and contribute decisively to the allosteric interaction networks. Based on our earlier studies [102], we elected to use a cutoff value of commute times 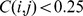 as a criterion for a residue pair 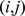 which are at a distance of 20 Å or greater to be considered as having an effective CP. We set this value as the threshold for distinguishing fast and effective communications. According to these criteria, allosterically communicating residues may have fast commute times despite their long-range structural separation.

We have previously determined that the Hsp90-N residues 81–95 and 121–140 (according to the residue numbering in PDB ID 2CG9) exhibited long-range communication with segments 574–580 and with the two C-terminal interface helices [102], [107]. The communication profiles obtained in the present work were consistent with these earlier studies, indicating that topography of the allosteric communication networks in Hsp90 can be adequately captured by the elastic network modeling. A similar pattern of communication propensities was obtained for key residues enabling allosteric regulation of the ATP-bound Hsp90 dimer. The effectively communicating clusters included the Hsp90-N residues (T22, A41, G81, G121, G170, 207-LVVT-210), the Hsp90-M residues (357-LIPE-360, 379-SRE-381, 426-KLG-428, 479-LKAVE-481) and the Hsp90-C residues (576-AAIRTG-581) (Figure 6A). Most of these residues lie close to the inter-domain boundaries and some clusters contain the critical catalytic residue R380 (379-SRE-381) and a regulatory switch point A-577 (576-AAIRTG-581) [113]. The structural arrangement of high and low stability residues comprising these communication clusters may allow for proper functional dynamics of the catalytically competent Hsp90 dimer. In the Hsp90-p23 complex, the reduced conformational mobility of the Hsp90-N and Hsp90-M regions affected some of these allosteric pathways and led to activation of alternative and arguably less efficient communication routes connecting the Hsp90-N domains with M-C boundaries through the clusters 351-TDEAE-355 and 426-KLG-428 (Figure 6B). Importantly, some of the communication networks connecting the Hsp90-M cluster 426-KLG-428 with the Hsp90-C regulatory region 576-AAIRTG-581 remained unperturbed in the Hsp90-p23 complex (Figure 6B).

According to our analysis, p23-mediated changes in the Hsp90 dynamics are associated with stabilization of the Hsp90-N dimer interactions that are supported by further consolidation of the N-M contacts. Based on presented results, these interactions may provide additional “molecular brakes” that could slow down an efficient transmission of the inter-domain allosteric signals. At the same time, the net effect on global motions of the chaperone may be rather marginal and transpire in cooperative movements of the Hsp90-N domains and p23 molecules acting as a “single body” connected with the hinge regions. In our interpretation, similarity of collective motions may reflect a common topography of the allosteric network, whereas selection and activation of specific communication routes seems to be cochaperone-dependent and potentially function as a “relay switch” of the conformational equilibrium. This distinctive dynamic signature of the Hsp90-p23 complex may be associated with the functional role of p23 that partially inhibits the ATPase cycle via trapping Hsp90 in a hydrolysis-competent state, which is likely to regulate the residence time of “highly demanding” clients favoring this specific form [19]–[23].

### Protein Docking and Structural Analysis of the Hsp90-Aha1 Complexes: The Role of the Aha1-N Domain

In this section, we describe the results of structural modeling of the Hsp90 complexes with the individual Aha1 domains and a complete Aha1 construct. Experimentally-guided docking using the HADDOCK approach [110] was used to assemble structures of the Hsp90-Aha1 complexes. MD-based optimization of the predicted models refined the intermolecular binding interfaces allowing for a detailed characterization of the Hsp90-Aha1 interactions. The results of protein docking and structural modeling were validated by a detailed comparison with the NMR data and biophysical experiments [66]–[69]. According to the recent evidence [69], [70], Aha1 may recognize and transiently bind to multiple forms of Hsp90, yet the thermodynamic preferences stabilize the formation of the Hsp90-Aha1 complexes with a closed form of the chaperone. To emulate this scenario of Aha1 binding and validate our docking approach, we adopted a HADDOCK protocol that simultaneously probed binding preferences of Aha1 with multiple and structurally different forms of Hsp90. The ensemble of multiple Hsp90 conformations used in docking runs was generated using previously reported all-atom MD simulations [107]. The protein ensemble included a total of 50 representative snapshots taken from MD simulations of the crystal structure of yeast Hsp90 (PDB ID 2CG9) [38]; the crystal structures of the bacterial homologue HtpG in an open free form (PDB ID 2IOQ) and an ADP-bound form (PDB ID 2IOP) [39], and the crystal structures of the mammalian Grp94 homologue in complexes with ADP (PDB ID 2O1V) and AMP-PNP (PDB ID 2O1U) [40].

**Figure 7:**
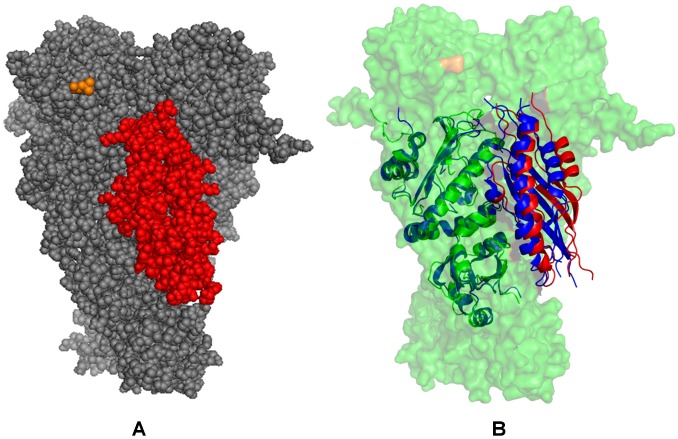
The Predicted Model of the Aha1-N Domain Complex with Hsp90 Dimer. The predicted low-energy model of the Aha1-N domain bound to the Hsp90 dimer is shown in (A) in a sphere-based protein representation. The Hsp90 dimer is shown in grey spheres, the docked Aha1-N domain is colored in red spheres. The best low-energy models of the Aha1-N complexes obtained in the initial analysis (panels A and D in Figure S1) were optimized in additional rounds of the HADDOCK and MD refinement. The intermolecular AIR was supplemented by the intramolecular constraints to preserve secondary structures in the Aha1-N during semi-flexible stages of HADDOCK refinement. These intramolecular interactions were formatted into a distance restraint file and uploaded to HADDOCK as unambiguous interaction restraints. (B) Superposition of the predicted structural model of the Aha1-N complex in the dimer with the crystal structure of the Aha1-N complex with the Hsp90-M (PDB ID 1USU) [66]. The docked Aha1-N domain is shown in red ribbons and the Hsp90-M domain of the chaperone dimer is in green ribbons. The crystal structure of the Aha1-N domain bound to the Hsp90-M domain is shown in blue ribbons. The structure of the Hsp90 dimer is enclosed in a 40% transparent molecular surface colored in green.

We began by performing docking simulations using only the crystal structure of the Aha1-N domain, since this domain was implicated as a main driver in stimulation of the ATPase activity [68], [69]. These simulations incorporated the experimental observation that binding of the Aha1-N domain with Hsp90 is nucleotide-independent, whereas the Aha1-C domain can interact with the Hsp90-N domains in the presence of ATP or AMP-PNP [66]. As a result, in docking of the Aha1-N domain the representative Hsp90 snapshots were extracted from all-atom MD simulations performed with the removed nucleotide [107]. The assembled sets of active and passive residues forming the AIR templates (Table S2) reflected the topology of the Hsp90-Aha1 assembly, namely the fact that the Aha1-N would primarily interact with the Hsp90-M [66]. We highlighted the focal points of docking simulations by analyzing the low energy solutions obtained with 5 different AIR sets. Irrespective of the composition of experimental restraints, docking results reproduced the energetic preferences of Aha1 to preferentially interact with the closed form of the Hsp90 dimer (Figure S1). The predicted Hsp90-Aha1 complex reproduced the interaction site of the Aha1-N from the crystal structure with the Hsp90-M and accurately mapped the binding interface with the Hsp90-N (Figure 7). Structural analysis of the docked solutions revealed a gradual consolidation of the Hsp90 interface with the Aha1-N as the HADDOCK-based score of the complexes improved (Figure S1). While less favorable docking solutions displayed minor packing defects, the predicted low-energy model accurately reproduced the overall topology of the Hsp90-Aha1 complex and the main interaction sites that were originally inferred in the NMR experiments [68]. Interestingly, a sheer number of incorporated experimental restraints did not translate into the improved structural model, which may be due to the increased stiffness of the underlying energy landscape that could hamper conformational sampling of the low-energy complexes. Somewhat unexpectedly, and yet quite instructively, the lowest energy complex with the Aha1-N was obtained by employing a rather “minimalistic” AIR template. In this case, the experimental restraints used in docking were extracted exclusively from the crystal structure of the Hsp90-M domain complex with the Aha1-N [66], where only hydrophobic residues contributing to the binding interface were chosen as active residues. The scatter graphs of the HADDOCK score and the intermolecular interface RMSD (Figure S2) of the docked conformations reflected important features of the binding energy landscape. A moderate correlation between energy scores and RMSDs in the main cluster of docked solutions may reflect the driving force favoring the native topology of the Hsp90-Aha1 complex. However, docked conformations near the native basin (within RMSD ∼2.0 Å from the lowest energy complex) could span a range of scores, reflecting structural plasticity of the interacting partners near the binding interface (Figure S2).

**Figure 8:**
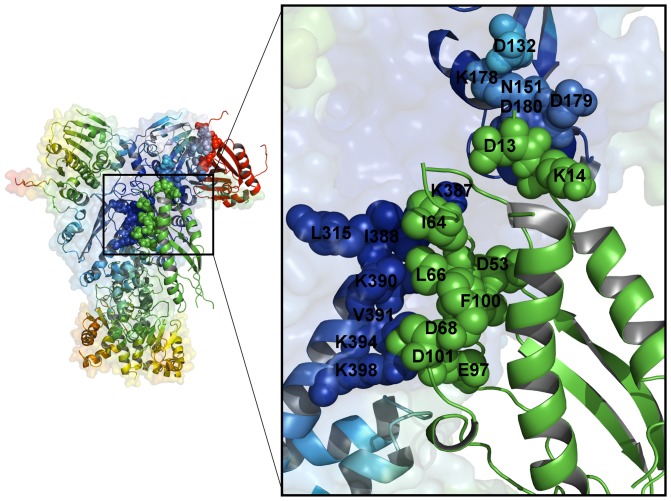
Structural and Dynamic Mapping of the Aha1-N Interactions with Hsp90. The predicted binding interface between the Aha1-N and the Hsp90 dimer is presented. (Left Panel) Structural distribution of conformational mobility in the Hsp90-Aha1 complex was obtained from the functional dynamics analysis. A ribbon-based protein representation is employed. The color gradient from blue to red indicates the decreasing structural rigidity of protein residues as in Figure 4. The Hsp90 dimer is also enveloped in a 50% transparent molecular surface. The Hsp90 residues from the intermolecular interface are colored according to their mobility (blue to light blue spheres). For clarity of presentation, the Aha1-N domain is shown in green ribbons, the Aha1-C domain in red ribbons. The Aha1-N and Aha1-C interfacial residues are shown respectively in green and red spheres. (Right Panel) A close-up of the Aha1-N binding interface. A ribbon-based protein representation with a 40% transparent molecular surface at the background is used. The interfacial Hsp90 residues are annotated and shown in spheres colored according to their mobility in the complex (from rigid/blue to flexible/red). The Aha1-N interfacial residues are shown in green spheres. Of notice, a central hydrophobic cluster formed by I64, L66 and F100 of the Aha1-N and L315, I388 and V391 from the Hsp90-M domains. A network of hydrogen between D53, D101, D68 and E97 from the Aha1-N and K387, K390, K394 and K398 of the Hsp90-M domains is recognized as a crucial stabilizing contributor of the Hsp90-Aha1 complex [66]. The predicted interacting residues in the Hsp90-N are D132, N151, T157, K178, D179, and D180.

MD-based refinement of the predicted docked complexes allowed for structural mapping of the interacting residues and characterization of the Aha1-N interactions with the Hsp90 dimer (Figure 8). The predicted model of the Hsp90-Aha1 complex reproduced structural stability of the central hydrophobic cluster formed by I64, L66 and F100 of the Aha1-N and L315, I388 and V391 from the Hsp90-M domains**.** A network of salt bridges formed between D53, D101, D68 and E97 from the Aha1-N and a group of lysine residues K387, K390, K394 and K398 of the Hsp90-M domains is recognized as a crucial stabilizing contributor of the Hsp90-Aha1 complex [66]. This important element of the intermolecular interface was accurately captured in docking simulations and remained stable during the MD refinement stage (Figure 8). In agreement with the NMR mapping [68], structural modeling also predicted a second interacting site formed between the Hsp90-N and the Aha1-N domains. This secondary interaction site is smaller and involves a group of the Aha1-N residues interacting with the portion of the ATP-binding pocket in Hsp90. The predicted interacting residues in the Hsp90-N (D132, N151, T157, K178, D179, and D180) exhibited chemical shift differences in the NMR analysis of the Hsp90-Aha1 interactions [68]. Importantly, this prediction was obtained using a minimalistic set of restraints that included only the Hsp90-M active residues. None of the Hsp90-N residues were entered into the AIR template and there was no any bias favoring the formation of these interactions.

### The Individual Domains of Aha1 Can Synergistically Interact with the Hsp90 Closed Dimer

Aha1-mediated stimulation of the Hsp90-ATPase activity requires binding of a complete Aha1 construct [68], [69]. The full-length human Aha1 consists of the Aha1-N domain (residues 1–162), a degenerate linker (residues 163–205) and the Aha1-C domain (residues 206–338) [68], [69]. To understand structural role and potential synergistic effects of the individual Aha1 domains in the regulatory mechanism we also carried out docking simulations of the Aha1-C domain with the Hsp90 dimer. The NMR experiments suggested a mechanism of “sequential” Aha1 binding to Hsp90, where the initial binding of the Aha1-N domain is critical for the initial stimulation of the ATPase activity and may trigger the recruitment of the Aha1-C domain that is required for consolidation of the Hsp90-Aha1 complex and the ATPase activation [68], [69]. The “sequential” mechanism was mimicked in our modeling experiments by also incorporating the experimental evidence that the interacting sites of the Aha1-N and Aha1-C domains are non-overlapping [68], [69]. By using the low-energy complexes of the Aha1-N domain with Hsp90 as starting points for docking of the Aha1-C domain, we assembled and refined the structure of the complete Hsp90-Aha1 complex. In docking simulations we used the solution structure of the human Aha1-C (PDB ID 1×53) and a homology model of yeast Aha1-C created based on the human NMR structure as adopted in the NMR studies [68].

**Figure 9:**
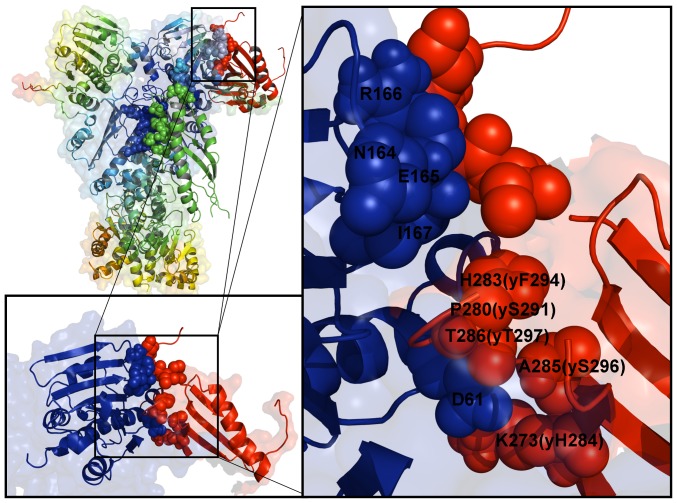
Structural and Dynamic Mapping of the Aha1-C Interactions with Hsp90. (Left Upper Panel) Structural distribution of conformational mobility in the Hsp90-Aha1 complex was obtained from the functional dynamics analysis. A ribbon-based protein representation with a background transparent surface view is employed. The Hsp90 residues from the intermolecular interface are colored according to their mobility (blue to light blue spheres). For clarity of presentation, the Aha1-N domain is shown in green ribbons, the Aha1-C domain in red ribbons. The Aha1-N and Aha1-C interfacial residues are shown respectively in green and red spheres. (Lower Left Panel) An overview of the Aha1-C binding interface. The Hsp90-N is shown in blue ribbons with the interfacial residues in blue spheres. The Aha1-C is shown in red ribbons and the interfacial residues are indicated by red spheres. (Right Panel) A detailed close-up of the Aha1-C interactions with the Hsp90-N domain. The interacting residues in the Hsp90-N included N164, E165, and R166 residues (shown in blue spheres). The interacting residues in the human Aha1-C are K273, P280, H283, A285, and T286 (shown in red spheres). The corresponding residues in the yeast Aha1-C are H284, S291, F294, S296, T297 are also annotated.

The predicted binding interface between the Aha1-C and the Hsp90-N domains was consistent with the NMR mapping [68] and mass spectrometry analysis [69]. The interacting residues in the Hsp90-N included N164, E165, and R166 residues, all of which experienced significant chemical shift changes in the NMR experiments [68]. We found that the most favorable binding mode involved a network of interactions formed by the Aha1-C residues from the β-sheet region of the cochaperone structure (Figure 9). The interacting residues in the human Aha1-C included a conserved residue stretch 279-WPEGHFAT-286 where H283, A285 T286 cooperate with K273 to establish the key interactions with the Hsp90-N. The respective cluster of interfacial residues in the yeast Aha1-C (290-WSAPFNST-297) featured F294, S296, and T297 that together with H284 formed the primary interacting contacts (Figure 9). This is also consistent with the biophysical cross-linking experiments which confirmed that the residues 276-FKSWPEGHFATTIL-289 from the Aha1-C would interact with the Hsp90-N domain [69]. Indeed, we observed that a subgroup of these residues 279-WPEGHFAT-286 along with K273 was involved in direct interactions with the Hsp90-N. Interestingly, K273 and residues 283-HFATTIL-289 were fully protected from covalent modifications under physiological conditions in solution [69]. Docking simulations also revealed a less favorable binding mode involving a small interacting loop on the opposite side of the Aha1-C domain (data not shown) formed by residues I292, G296, E297 in the human Aha1-C (yeast residues H303, F308, H309, and E310). According to the experimental data, these residues experienced less significant changes in the chemical shifts [68]. Hence, the agreement between computational and experimental data could also capture this more subtle effect and confirmed the primary interaction site between the Aha1-C and the Hsp90-N domains.

The binding interface formed by the Aha1-N is rather extensive and involves interactions with conserved residues from multiple domains of Hsp90 (Figure 8). On the other hand, the interface formed between the Aha1-C and the Hsp90-N is localized, much smaller and more dynamic as the contributing residues from the interacting partners belong to the peripheral flexible regions (Figure 9). These structural observations underscore the notion that the Aha1-N may be essential for primary stimulation of the ATPase activity, whereas the C-terminal domain of Aha1 would likely to play a supporting role by enhancing the Aha1-N interactions to the Hsp90-N [68], [69]. According to the predicted structural models (Figures 8, 9), binding of the Aha1-C could have a synergistic effect by “stitching” the Hsp90-N domain of the interacting monomer with both Aha1 domains. The rigidified structural environment of the Aha1-interacting Hsp90-N domain can then facilitate the formation of a closed dimerized form of the chaperone. In this scenario, the Aha1-C interactions with the Hsp90 dimer would not compromise and may further stabilize structural assembly of Hsp90 with the Aha1-N domain. The analysis of Aha1-mediated interactions with Hsp90 suggested that the domains of Aha1 may act cooperatively to direct stochastically-driven cycle towards a closed state to maximize the stimulation of ATP hydrolysis [68]–[70].

### Dynamic Fingerprints of the Hsp90-Aha1 Complexes: Allosteric Modulation of Collective Motions

**Figure 10:**
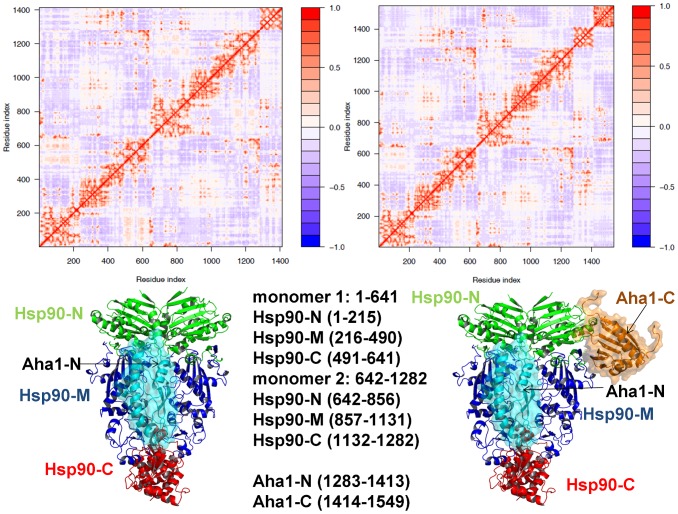
Analysis of Correlated Motions in the Hsp90-Aha1 Complexes. The cross-correlation matrices of residue fluctuations computed along the low frequency modes in the Hsp90 complex with the Aha1-N domain (A) and in the complete Hsp90-Aha1 complex (B). The matrix was calculated using the results of MD-based refinement and NMA of the predicted structures for the bound Aha1-N domain (A) and the complete Aha1 molecule (B). The original residue numbering in the crystal structure of yeast Hsp90 (PDB ID 2CG9) was converted to a consecutive numbering in the cross-correlation matrix of the Hsp90 dimer as described in the caption of Figure 5. The monomer 1 includes residues 1–641 and the monomer 2 consists of residues 642–1282. In the monomer 1: Hsp90-N (residues 1–215), Hsp90-M (residues 216–490) and Hsp90-C (residues 491–641). In the monomer 2: Hsp90-N (residues 642–856), Hsp90-M (residues 857–1131), and Hsp90-C (residues 1132–1282). The Aha1-N domain includes residues 1283–1413 and the Aha1-C domain residues are in the range 1414–1549 respectively.

Analysis of principal collective motions in Hsp90 has identified functional motifs, including the inter-domain hinge regions that may act collectively as regulators of the allosteric communications and cochaperone binding [107]. We proposed that Aha1-based regulation of the ATPase activity may be associated with modulation of collective motions via targeted binding to the regulatory hinge motifs. This could alter functional dynamics of the chaperone and allow for sequestration of a specific N-dimerized closed state of Hsp90. The MD-refined structural models of the Hsp90-Aha1 complex were subjected to the GNM analysis to probe the differential effect of the Aha1 domains on functional motions of Hsp90. The distribution of conformational mobility and the cross-correlation maps of protein residue fluctuations were computed along the low frequency modes. The collective movements of Hsp90 are determined by the topology of the homodimer architecture and cannot be significantly altered upon binding. Nevertheless, we noticed important signs of Aha1-mediated alterations in the collective motions that could allow for modulation of the chaperone activity. Importantly, sequential binding of the Aha1-N (Figure 10A) and the complete Aha1 molecule (Figure 10B) may lead to the progressive weakening of concerted motions affecting both the intra- and the inter-monomer couplings. In particular, the positive intra-monomer correlations between the N-terminal and C-terminal domains as well as the positive inter-monomer correlations between the M-domains were compromised in the Hsp90 complexes with Aha1. A subtle decrease in the anti-correlated inter-monomer motions of the N-terminal and C-terminal domains could be also noticed.

Interestingly, the motions of the Aha1-N domain(residues 1283–1413) are positively correlated with the Hsp90-M domain (residues 216–490) of the interacting monomer 1, but uncoupled from the Hsp90-M domain of the other monomer (residues 857–1131). Hence, Aha1-N binding may suppress the inter-monomer correlations of the M-domains that are known to be associated with opening-closing movements of the chaperone [107]. In the asymmetric binding mode, the Aha1-N domain can interact with both the Hsp90-M of the first monomer 1 and the Hsp90-N of the second monomer, whereas the Ana1-C domain mainly contacts only the Hsp90-N of the second monomer (Figures 8, 9). A corresponding moderate effect inflicted by Aha1-C on collective motions may be a reflection of its supporting role in stimulation of the ATPase activity [68], [69]. Nonetheless, asymmetric binding of the complete Aha1 construct could give rise to the positive correlated motions of the Aha1-C with the Hsp90-N of the second monomer, while decreasing correlations with the Hsp90-N of the first monomer (Figure 10B). In the Hsp90-Aha1 complex, a synergistic binding of the Aha1 domains with the Hsp90-N (residues 642–856) is responsible for improved positive correlations of Aha1 with this chaperone domain. Overall, the analysis of correlated motions indicated that asymmetric binding mode of the Aha1 domains may partially uncouple the concerted inter-monomer motions in the Hsp90-N and Hsp90-M domains. These factors may hinder global cooperative movements of Hsp90 that are required for the normal progression of conformational changes in the ATPase cycle.

Functional coupling of structurally stable and mobile residues in the regulatory hinge regions situated at the inter-domain boundaries is critical to enable collective motions of Hsp90. It is instructive to analyze the pattern of correlated motions in these regions induced by Aha1 binding. In the unbound and nucleotide-free form of Hsp90, the Hsp90-M domains of the monomer 1 (residues 216–490) and monomer 2 (residues 857–1131) were positively correlated (Figure 5A). In particular, the N-M inter-domain hinge residues 319–328 of the first monomer (corresponding to the residues 374-LPLNLSREML-383 in the crystal structure of yeast Hsp90 [38]) exhibited mostly positive correlations with the Hsp90-N and Hsp90-M of the same monomer and anti-correlated motions with the Hsp90-N of the other monomer. During “sequential” binding of Aha1 domains these functional motifs become progressively insulated from the allosteric interaction networks and experienced a reduction in the long-range correlations with the N-terminal and M-domain regions (Figure 10). These changes may be sufficient to partially impair the hinge movements required for conformational transitions during the Hsp90-ATPase cycle.

Structural environment of the second helix (residues 386-SKNIKLGVHE357-395) in three-helix bundle from the monomer 1, including the M-C hinge residues 390-KLGVHE-395 (where the latter corresponds to the hinge residues 426-43 in the crystal structure numbering) is subjected to positive correlated motions with the Hsp90-M and Hsp90-C domains of both monomers, which reflects its role in coordinating the long-range inter-domain movements (Figure 10). In the Hsp90-Aha1 complex, this region may become partially uncoupled from long-range interactions as judged by a moderate reduction in the extent of correlated motions with other chaperone regions (Figure 10). According to our analysis, Aha1-mediated alteration of correlated motions near the regulatory hinges may affect the equilibrium fluctuations between functional states. In this scenario, targeted binding of Aha1 to the hinge regions could modify the global distribution of the conformational ensemble and modulate normal progression of the ATPase cycle. The presented arguments support an arguably broader role of hinge motifs in mechanisms of binding-induced allosteric transitions, according to which hinges often serve as non-redundant functional nodes with unique mobility profiles [114], [115].

### Functional Dynamics of the Hsp90-Aha1 Complexes and Allosteric Effects of Binding: Computation and Experiment

**Figure 11:**
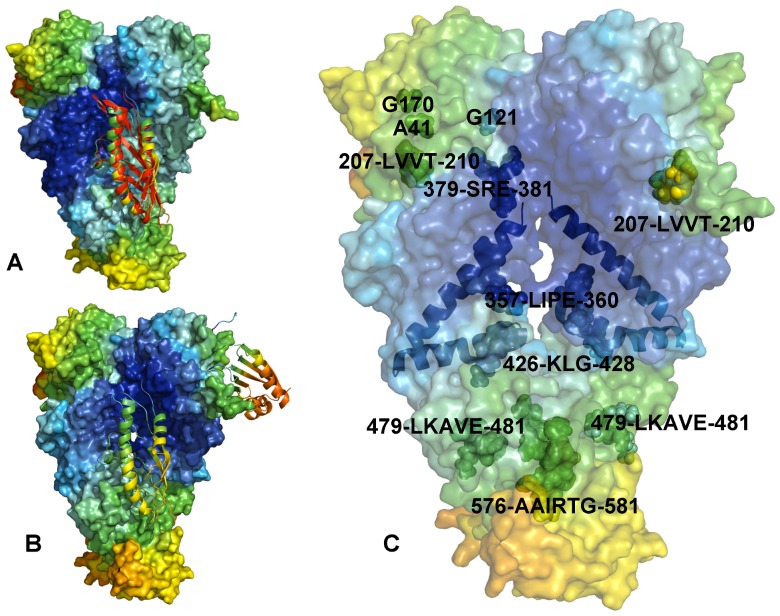
The Allosterically Communicating Residues in the Hsp90-Aha1 Complex. The functional dynamics profiles of the Hsp90-Aha1 complexes with the Aha1-N domain (A) and the complete Aha1 molecule (B). A ribbon-based representation of the Aha1 domains is combined with a surface view of the Hsp90 dimer. The color gradient from blue to red indicates the decreasing structural rigidity of protein residues as in previous figures. (C) The distribution of allosterically in the context of the functional dynamics profile. The location and identities of allosterically communicating residues are annotated in spheres colored according to their respective mobility level. The crystal structure of yeast Hsp90 is enveloped in a 50% transparent molecular surface to show the location of the allosterically communicating residues. The three-helix bundle that links the inter-domain regions is shown in ribbon representation.

**Figure 12:**
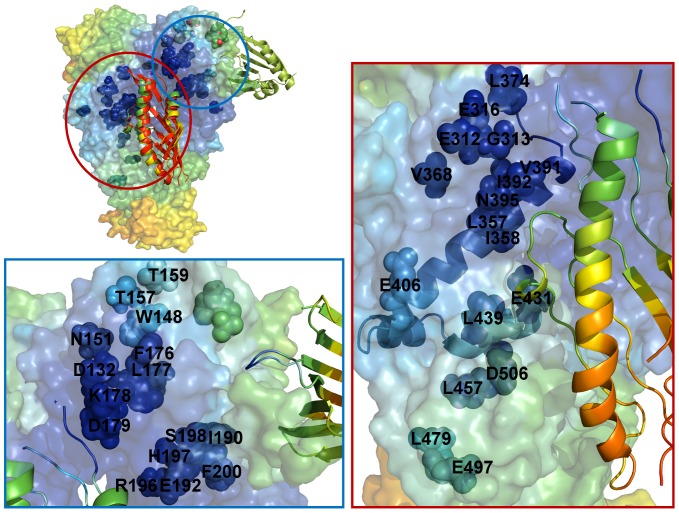
The Aha1-Mediated Effect on Conformational Mobility of Hsp90. (Left Upper Panel) Conformational mobility map of the Hsp90-Aha1 interactions with the Aha1-N and Aha1-C domains was compared with the NMR experiments [68], [69]. The annotated Hsp90 regions (in spheres colored according to their mobility) correspond to the residues that experienced significant chemical shift perturbations upon Aha1 binding. (Right Panel) A close-up of the predicted Aha1-N binding interface with the Hsp90-M and Hsp90-N domains. The Aha1-N domain is shown in ribbon representation. The Aha1-N binding region involves residues from the three-helix bundle (shown in ribbon representation). Binding of the Aha1-N domain could affect structural stability of the Hsp90-M residues located near the N-M hinge region including E312, G313, E316, L357, I358, L374, V368, V391, I392, N395 (Left Lower Panel) A close-up of the predicted Aha1-C binding interface with the Hsp90-N domain. The Ana1-C is shown in ribbon representation. The conformational mobility profiles of the Hsp90 residues that were affected by the Aha1-N and Aha1-C binding are annotated in colored spheres. The allosteric effect of Aha1 binding can rigidify the Hsp90-N residues W148, T159, N164, E165, F176, L177, K178, D179, H197 and F200.

In this section we characterized the effect of Aha1 binding on functional dynamics and distribution of allosteric communication pathways in Hsp90. Of particular interest was the analysis of Aha1-induced changes in the distribution and allocation of structurally stable and conformationally flexible residues. Complex biological systems can integrate the adaptability and robustness by maintaining the appropriate balance between structural rigidity and flexibility. We addressed the question whether Aha1-based modulation of the Hsp90 activity is associated with dynamic changes in structural rigidity and flexibility that are crucial for proper functioning of the chaperone. Structural mobility profiles of the Hsp90-Aha1 complexes revealed a considerable strengthening of structural rigidity around binding interfaces (Figures 11, 12). Moreover, regions that are proximal to the three-helix bundle and intimately involved in the inter-domain interactions become largely immobilized in the Hsp90-Aha1 complex (Figure 11) as compared to the unbound form of Hsp90 (Figure 4A). The allosteric effect of the Aha1-N interactions manifested in the increased structural stability spread across all domains of the interacting Hsp90 monomer (Figure 11A). According to our findings, Aha1 domains could synergistically strengthen the N-M interactions and completely rigidify structural environment of the Hsp90-N in the interacting monomer that becomes primed for the formation of a closed dimerized state. At the same time, conformational mobility of the opposite Hsp90-N domain could progressively increase with the addition of the Aha1-C domain (Figure 11B). This may transpire in partial decoupling of the Hsp90-N domains movements. Collectively, these factors may trigger a dislocation and imbalance of structurally rigid and conformationally flexible residues across the entire homodimer.

We also analyzed changes in the distribution of allosterically communicating residues in the Hsp90-Aha1 complex in the context of the functional dynamics profile (Figure 11C). As was shown in the previous sections, the appropriate balance of high and low stability is an important feature of allosterically connected residue clusters in the ATP-bound Hsp90 dimer. In the Hsp90-Aha1 complex, the increased homogeneity in structural rigidity of the Hsp90-N and Hsp90-M domains could affect communication capabilities of some residue clusters (Figure 11C). The observed “deactivation” of allosteric communication routes in the Hsp90-N domain of the second monomer may result from its uniform structural immobilization by the Aha1 domains. Additionally, some effectively communicating residues in the Aha1-interacting M-domain may be also uncoupled from the allosteric networks. Indeed, a dense communication pathway connecting the Hsp90-M hinge regions in one of the monomers through a three-helix bundle may become sparse in the Hsp90-Aha1 complex as some contributing residues could be “deactivated” by failing to hit the threshold criterion for effective communicators (Figure 11C). In particular, functionally important for dimerization T22 may be bypassed in the modified communication pathways. It is possible that Aha1-mediated allosteric interactions stimulating the ATPase activity may potentially offset this loss by “turning on” alternative communication routes. Indeed, two-hybrid analysis of yeast Hsp90 [116] and biochemical studies of Hsp90 binding [117], [118] have shown that the ATPase enhancement by Aha1 can persist in the Hsp90-T22E mutant, suggesting that Aha1 could still enforce an N-terminal dimerized state and compensate the loss of the ATPase activity. Mutational analysis has similarly demonstrated the ATPase activity of the Hsp90-T22I mutant could be unaffected by the decreased expression of Aha1 [119]. We then compared the conformational mobility map of the Hsp90-Aha1 complex with the NMR and biophysical experiments [68], [69] by focusing on the residues that experienced significant chemical shift perturbations upon Aha1 binding. The titration of the Aha1-N domain with the Hsp90 dimer induced chemical shift changes in the N-M hinge residues 374-LPLNLSREML-383 as well as in the group of residues 390-KVIRKNIVKK-399 [68]. In agreement with the NMR data, we observed that binding of the Aha1-N domain could affect structural stability of the Hsp90-M residues located near the N-M hinge region including E312, G313, E316, L357, I358, L374, V368, V391, I392, N395 (Figure 12). Importantly, the inter-monomer interactions formed by some of these Hsp90-M residues (L372, L374, and R376) with the Hsp90-N residues of the other monomer (T22, V23, Y24) are critical for regulation and can facilitate the ATP hydrolysis [120]. According to our results, Aha1-mediated changes in the dynamic profile of Hsp90 are largely associated with the increased structural stability and consolidation of the inter-monomer N-M interactions (Figure 12). The increased stability of the Hsp90-M residues E431, L439, L457, L479, E497 and D506 is consistent with the observed chemical shifts in these regions [68]. The impact of the Aha1-C interactions on the dynamic profile of Hsp90 could also extend beyond the interacting site and rigidify the Hsp90-N residues W148, T159, N164, E165, F176, L177, K178, D179, H197 and F200 (Figure 12). This is consistent with the NMR mapping that identified significant chemical shift changes in clusters of residues 148–159, 175–180, and 192–200 [68].

**Figure 13:**
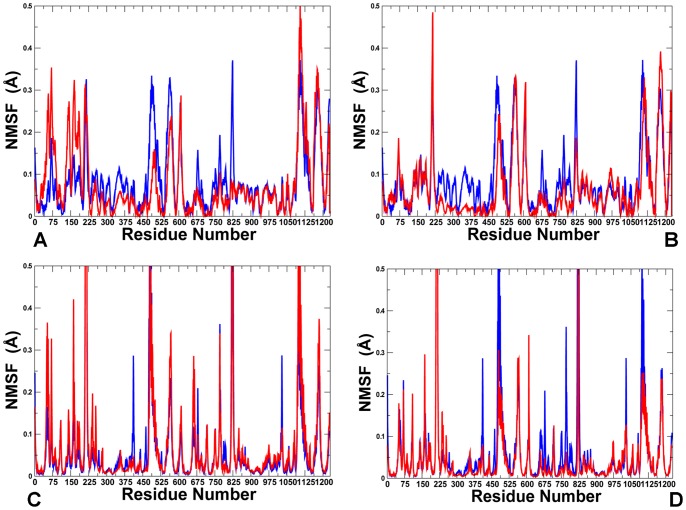
The Residue-Based Fluctuation Profiles of Hsp90: The Effect of the Aha1 Domains. The NMSF profiles of the Hsp90 residues were computed along the lowest frequency mode (A, B) and by averaging the residues fluctuations over 10 low frequency modes (C, D). The NMSF profiles of the unbound Hsp90 (in blue) and in the bound Hsp90 form (in red) are shown for the predicted complex with the Aha1-N domain (A,C) and the complex with a complete Aha1 molecule (B,D). The differences in the NMSF profile of Hsp90 induced by Aha1 binding were spread through multiple chaperone domains. The low-frequency motions of the inter-domain regions around hinge motifs are considerably curtailed. Functional movements of the Hsp90-N in the first monomer towards structurally rigid Hsp90-N in the second monomer could be induced by Aha1 binding. The crystal structure residue numbering was converted to a consecutive numbering as in Figure 3. The annotation for the monomer 1 of Hsp90 is the following: Hsp90-N (residues 1–215), Hsp90-M (residues 216–471) and Hsp90-C (residues 472–609). The annotation for the monomer 2 of Hsp90: Hsp90-N (residues 610–824), Hsp90-M (residues 825–1089), and Hsp90-C (residues 1090–1227).

The presented results argued that the Aha1-mediated changes in collective motions and communication pathways could accelerate structural rearrangements that would help to trap Hsp90 in the N-terminal dimerized closed form. To identify and characterize these structural changes, we analyzed functional motions in the Hsp90-Aha1 complex that are represented by the normalized mean square residue fluctuations (NMSF) along the low frequency modes. In these distributions, stationary points are typically attributed to either flexible sites (the distribution maxima) or globally anchored regions which may act as hinges (the distribution minima). In this model, structurally stable residues with correlated thermal fluctuations that correspond to stationary points of the NMSF function can form clusters of allosterically communicating residues [81], [82], [107]. Importantly, not only structurally rigid but also conformationally mobile residues that exhibit a concerted change in their fluctuations would be recognized in this model as effectively communicating and assigned to the allosteric interaction clusters. The differences in the NMSF profile of Hsp90 induced by the Aha1-N interactions were distributed across all chaperone domains (Figure 13A). Most notably, one could observe the dampened fluctuations of the Hsp90-M (residues 216–490) and Hsp90-C (residues 491–641) domains of the Aha1-N interacting monomer as well as the Hsp90-M of the opposite monomer (residues 857–1131). These effects were further amplified by the Aha1-C interactions, particularly in curtailing the low-frequency motions of the inter-domain regions around hinge motifs (Figures 13B). An interesting finding from this analysis is an evidence of large functional movements of the Hsp90-N in the first monomer (residues 1–215) towards its structurally rigid counterpart from the second monomer (residues 642–856), that is completely immobilized by the interactions with the domains of Aha1. Importantly, this trend persisted when functional motions were computed by averaging the residues fluctuations over 10 low frequency modes (Figures 13 C, D). This could reflect the dominant character of these highly specific movements of the Hsp90-N domains that are induced by Aha1 binding. We argue that these functional motions may catalyze structural and dynamic changes that would increase the probability of bringing together the N-terminal domains and contribute to the formation of a closed dimerized state.

One of the basic functions of Aha1 is to destabilize the interactions of the catalytic loop with the Hsp90-M loop and release the catalytic arginine to a partially closed active orientation [68], [69]. According to our results, Aha1 may also accelerate slow equilibrium fluctuations of Hsp90 by effectively “freezing” the interacting Hsp90-N domain in a closed dimerized state while promoting large functional movements of the second Hsp90-N domain. This may reflect another important “dynamic” function of Aha1 as an activator of the ATPase activity. The proposed model is also consistent with the recent revelations suggesting that a dynamically regulated association of the N-terminal domains in the Hsp90-Aha1 complex could favor a partially closed state of Hsp90 and leave the nucleotide binding site accessible for ATP exchange before the chaperone is finally trapped in a hydrolysis-competent state [70].

### Protein Structure Network Analysis of the Hsp90 Complexes: The Effect of Cochaperones on Conformational Stability of Hsp90

**Figure 14:**
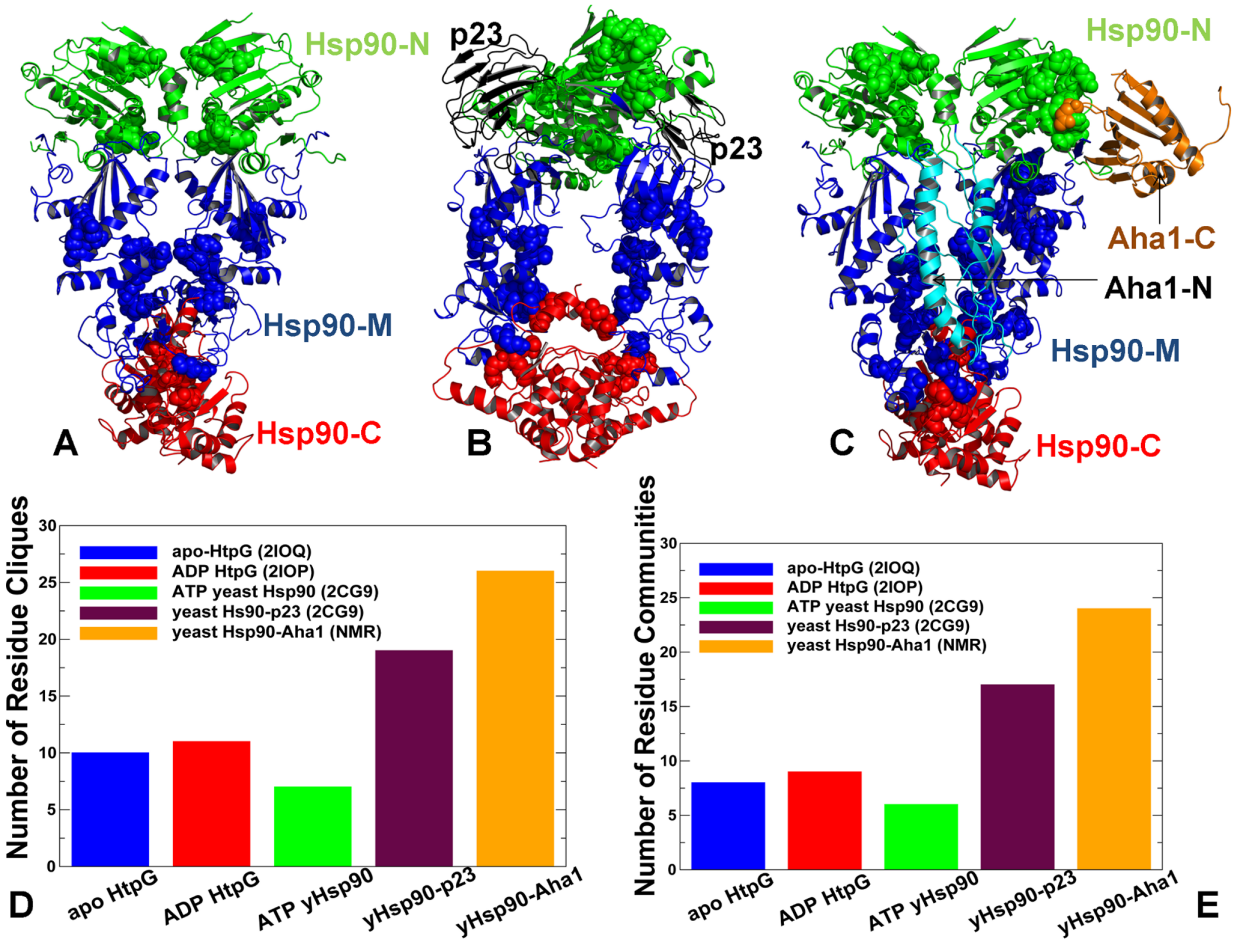
Protein Structure Network Analysis of the Hsp90 Complexes. The distribution of structural communities in the cochaperone-free ATP-bound Hsp90 (A), the Hsp90-p23 complex (B) and the Hsp90-Aha1 complex (C). The Hsp90 structures are shown in ribbon representation and colored according to their domain nomenclature : Hsp90-N in green, Hsp90-M in blue, and Hsp90-C in red. The Hsp90 residues that constitute structural communities are shown in spheres and colored according to their respective domains. The displayed networks correspond to structurally stable communities that remained intact in more than 75% of the simulation snapshots. The increased consolidation and integration of small communities into larger highly connected networks could be observed in the Hsp90-p23 (B) and Hsp90-Aha1 complexes (C). The number of residues-based cliques (D) and communities (E) in the Hsp90 structures obtained by averaging these parameters over MD trajectories of the crystal structure of yeast Hsp90 (PDB ID 2CG9) [38]; the crystal structures of the bacterial homologue HtpG in an open free form (PDB ID 2IOQ); an ADP-bound form (PDB ID 2IOP) [39], and MD refinement trajectories of the Hsp90-p23 and Hsp90-Aha1 complexes.

We supplemented functional dynamics mapping of the Hsp90-cochaperone complexes with a graph-based network analysis to identify functionally important residues that are responsible for both global motions and structural stability of the Hsp90 complexes. Using various network parameters such as hubs, cliques and communities [93]–[98], we quantified the effect of Aha1 and p23 binding on distribution and reorganization of structurally stable residue networks (Figure 14 A–C). By mapping network parameters onto functional dynamics profiles we also characterized the role of cochaperones in modulating balance between structural rigidity and flexibility. A community size is determined by the number of constituent cliques and is typically proportional to the protein packing density [93]–[100]. The protein structure network parameters such as cliques and communities could provide a simple metric for estimation changes in structural stability of Hsp90 upon cochaperone binding. A similar and rather moderate number of cliques and communities in the cochaperone-unbound Hsp90 may reflect the experimental evidence that conformational states of Hsp90 are thermodynamically only marginally stable. Consequently, binding of cochaperones and client proteins can readily shift the conformational equilibrium and stabilize a specific functional state by applying only a small amount of binding energy. We have observed that structurally stable communities in the Hsp90 structures are fairly conserved and assembled near the three-helix bundle that coordinates motions of the regulatory hinges (Figure 14A–C). The residues from the inter-domain boundaries belong to structurally stable communities in all studied Hsp90 structures suggesting that conservation of the inter-domain rigidity may be a universal property of the chaperone states. Mapping of structurally conserved networks in the ATP-bound Hsp90 revealed a moderate number of local communities that are spatially separated (Figure 14A, E). A certain degree of segregation between structurally stable regions may reflect the functional requirement for mixing rigidity and flexibility in the native chaperone to assure proper execution of conformational transitions during progression of the Hsp90-ATPase cycle.

Among structurally stable communities observed in the ATP-bound Hsp90 structure (Figure 14A) is a group of residues (E406, K307, and E402) situated at the end of the long helix 1 in the three helix bundle. Interestingly, structural environment of the inter-domain M-C hinge (residues 426-KLGVHE-431) includes a number of structurally stable residues. Indeed, according to our analysis, local structural communities (W361, L427, E431) and (K426, D503, D506) can contribute to conformational stability of the ATP-bound chaperone (Figure 14A). Another noticeable structural community is situated in the Hsp90-C domain (R579, R526, and E614). In addition, these regions of high structural stability could partially overlap with the networks of effectively communicating residues (426-KLG-428 and 576-AAIRTG-581) (Figure 4A). These regions belong to the inter-domain boundaries and locate in the proximity of a regulatory switch point A-577 [113]. Some of these residues (for instance K426 and R579) are shared by many structurally rigid communities and belong to structural stability hubs of Hsp90 (Figure 14A–C).

Strikingly, cochaperone binding can produce an appreciable increase in the number of cliques (Figure 14D) and communities (Figure 14E) in the Hsp90 structure. The network analysis identified the important residues that could contribute to strengthening of the inter-domain N-M and M-C interactions and allosteric connections in the Hsp90-p23 complex (Figure 14B). These interactions are required to stabilize a rather peculiar “three-point interaction” mode where each p23 molecule makes contact with two Hsp90-N domains and a large interface of one Hsp90-M domain. The reorganization of structurally stable residue networks upon p23 binding leads to the merger of small communities into larger highly connected networks. Furthermore, the number of communities at the inter-domain interfaces grows and the rigidity could be partially transmitted between the domains. Indeed, by tracing the connections between smaller communities, we could identify a network of structurally stable residues that could connect all three chaperone domains (Figure 14B). This structurally stable frame of the Hsp90-p23 complex could be formed as a result of the increased connectivity between local Hsp90-N and Hsp90-M communities ((Y293,Y299,K366), (E406,K307,E402), (W361,L427,E431), (K426,D503,D506), (D506,Y445,L501)) reaching out to the network of consecutive Hsp90-C communities (E507,N588,I592), (R579,K526,E614), (D527,D523,R579), (R579,E614,K570). These structurally stable networks may a primary driver for stabilization of the inter-domain interactions and the closed Hsp90 dimer.

Binding of Aha1 resulted in even a greater number of communities that are spread across all domains of the Hsp90 structure. A similar consolidation of smaller communities and the markedly enhanced stabilization of the inter-domain interactions could be seen in the Hsp90-Aha1 complex (Figure 14C). These structurally stable communities could form a dense interaction network linking the N-M and M-C interfaces via a group of interconnected clusters (W277,R347,D370), (Y293,D299,K366), (K335,K338,E360), (W361,L427,E431), (K426,D503,D506), (F510,L521,Y473), (L488,R579,D523), and (R579,D523,D527). Our analysis indicated that there are a significant number of common structurally stable communities that contribute to stabilization of both cochaperone-free ATP-bound Hsp90 as well as the Hsp90-23 and Hsp90-Aha1 complexes. Some of the residues participating in cliques and communities could partly overlap with allosteric communication pathways residing near the inter-domain boundaries. These observations support the evidence that the integrity of the domain−domain interactions is critical for both structural stability and allosteric signaling of the Hsp90 chaperone. A comparison of the network parameters among the Hsp90-cochaperone complexes indicated that cochaperone-induced conformational changes and seemingly minor variations in structures may give rise to the enhanced structural stability sufficient to modulate conformational equilibrium of the Hsp90 chaperone. The increased structural rigidity of the Hsp90 complexes and re-organization of the community maps near the hinge regions and binding interfaces underlies the role of cochaperones in modulating conformational transitions and stabilizing specific states of Hsp90. According to our results, a number functionally important residues may control collective movements, participate in allosteric communications and be responsible for structural stability of the Hsp90 complexes.

Although computational approaches may be still limited in their ability to fully capture atomistic details of this sophisticated regulatory machinery, we offer a plausible model that is consistent with a broad range of experiments and could reconcile several existing mechanisms of chaperone regulation. Our results suggest that cochaperone-based modulation of the ATPase activity is not merely a binary switch but rather a “dynamic continuum” of intermediate states exhibiting varying degrees of “activity/inactivity”. Following this scenario, allosteric binding of cochaperones may engineer spatially and temporally controlled changes in the conformational equilibrium that would direct the progression of the ATPase cycle. The multiple layers and redundancies in regulatory mechanisms of Hsp90 can be provided not only through recruitment of cochaperones but also via posttranslational modifications. A series of tyrosine phosphorylation events appeared to be sufficient to recruit various cochaperones, including Aha1, to the Hsp90 system and provide certain directionality to the chaperone cycle [121]. By targeting communication switch points, dynamic phosphorylation of Hsp90 can affect the conformational dynamics and regulate chaperone binding during late stages of the ATPase cycle [122]. Further understanding of a “cross-talk” between dynamics of cochaperone binding and posttranslational modifications may provide detailed clues to conformational switches turning the stochastic ATPase cycle into an optimized flow of structural changes required for client specificity.

### Cochaperone-Based Modulation of Conformational Flexibility in Hsp90: The Local Frustration Analysis

**Figure 15:**
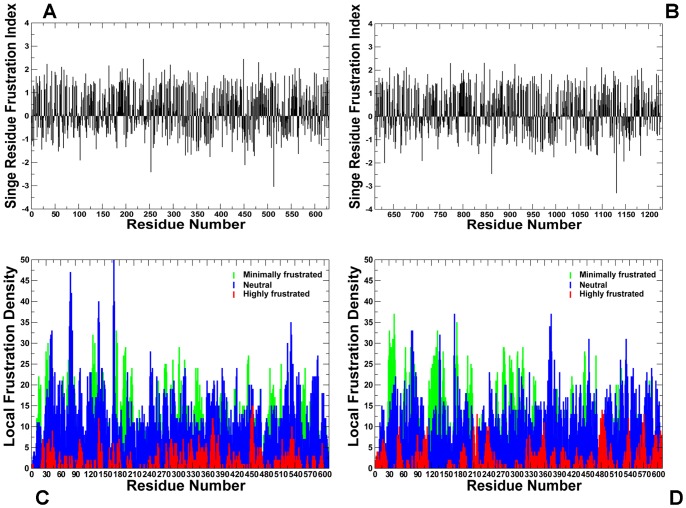
The Local Frustration Profiles of Hsp90. The residue-based configurational frustration index values are shown for the ATP-bound, cochaperone-free form of the Hsp90 dimer: monomer 1 (A) and monomer 2 (B). The integrated analysis the configurational frustration at the contact level is shown in (C, D). The local frustration density counts the number of contacts in each of the frustration categories within 5 Å from a given residue, i.e. how many contacts of a given residue are minimally frustrated (green), neutral (blue) or highly frustrated (red). The frustration density is represented in terms of the total numbers in each frustration category rather than the fraction of contacts in each class. (C) The local frustration density of the cochaperone-free (C) and Aha1-complexed forms of Hsp90 (D).

According to the energy landscape theory [123]–[129], allosterically regulated proteins can be characterized by a dynamic coupling between structurally rigid (minimally frustrated) and conformationally flexible (locally frustrated) regions [123]–[125]. We proposed that local frustration in the Hsp90 chaperone may arise from the requirements to adapt conformational dynamics for specific functions, including binding to cochaperones. To quantify local frustration we used the configurational frustration index that computes local stabilization for an individual native pair with respect to a set of structural decoys generated by perturbing both the identities and location of the interacting amino acids [126], [127]. The configuration frustration index classified high and low stability regions of the Hsp90 according to their frustration level. According to this metric, the ATP-bound active form of Hsp90 may present a “dynamic mix” of mostly unfrustrated and neutral residues, yet marked by a significant number of spikes corresponding to the locally frustrated residues (Figure 15 A, B). Interestingly, the local frustration analysis of the Hsp90 dimer is fully consistent with a broad survey of allosteric proteins [126], [127] suggesting that the majority of interactions are neutral (∼50%) or minimally frustrated (∼30–40%) and only ∼10% of the total contacts may be considered to be “highly frustrated”. The obtained distribution reflected the structural architecture of the chaperone, suggesting that conformationally mobile residues may often be immediately adjacent and form interacting clusters with their structurally rigid neighbors.

We also characterized cochaperone-induced differences in the distribution and allocation of locally frustrated residues (Figures 15 C, D). The patches of locally frustrated residues were found across all three domains of the ATP-bound Hsp90 dimer, including residues 319–328 from the N-M hinge region and residues 371–376 of the M-C secondary hinge (Figure 15C) (corresponding to the regions 374-LPLNLSREML-383 and 426-KLGVHE-431 respectively in the crystal structure 2CG9). Targeted modulation of the local frustration profiles in the Hsp90-Aha1 complex (Figure 15D) is notable in the Hsp90-N and Hsp90-M domain regions, particularly near the N-M hinge region, where the local frustration was reduced due to stabilizing interactions with Aha1 in this region (Figure 15D). In the Aha1-interacting regions the frustration level of the Hsp90 interface residues could be lowered to the levels of the interior residues seen in the ATP-bound Hsp90 dimer. These findings are reminiscent of the earlier conclusions reached in the analysis of single domain proteins [126]–[129]. However, the residues near the M-C inter-domain boundary could retain a considerable degree of local frustration required to modulate conformational changes in this region. One could notice a significant presence of locally frustrated sites corresponding to the loops in the Hsp90-C domain (residues 472–609). However, the regulatory switch region 512–526 in the Hsp90-C domain (corresponding to 576-AAIRTG-581 in the crystal structure) is minimally frustrated in both the cochaperone-free and Aha1-complexed forms of Hsp90 (Figures 15 C, D). By expanding previous analyses [126]–[129], we found that local frustration in the interacting sites could not be completely removed in the Hsp90-Aha1 complex, but was rather delicately attenuated. In general, we observed that Aha1 binding may elicit long-range changes in the allocation of highly frustrated residues and consolidate locally frustrated residues into sparsely distributed clusters (Figure 15D). We found that frustrated sites were not randomly scattered on the protein surface or uniformly distributed in the Hsp90 domains. Importantly, locally frustrated clusters could partly overlap with structurally rigid segments involved in allosteric interactions and collocate with the hinge regions directly involved in conformational changes associated with the chaperone function. The observed structural distribution of locally frustrated sites in Hsp90 may provide initiation points for global allosteric transitions via local rearrangements near hinge points. According to our findings, Aha1-mediated interactions could modulate the recruitment of locally frustrated residues to the minimally frustrated rigid clusters. The adjusted structural arrangement of high and low stability residues near communication clusters of the Hsp90-N and Hsp90-M domains may be thus potentially exploited to “fine-tune” functional movements and allosteric communications facilitating the formation of a closed dimerized state. The results suggest that the interplay of high and low stability residues in Hsp90 is quite dynamic, intimate and often involves functionally important for allosteric interactions regions. Structurally stable residues near regulatory hinge sites correspond to the minimally frustrated anchor sites, while the neighbouring flexible residues may be locally frustrated and prone to dynamic modulation by the cochaperones. The balance between structural rigidity and plasticity underlies the stochastic nature of conformational transitions in yeast Hsp90 [54]. Cochaperone-induced switches in the “frustration status” around pivotal hinge sites may alter this balance and result in allosteric structural changes that would shift the conformational equilibrium. In this mechanism, dynamic changes induced by cochaperones in the “rigidity-plasticity footprint” could promote thermodynamic stabilization of a closed dimer and increase the energetic barriers to prolong the residence time of this functional state. Our analysis offers a plausible mechanistic picture that may contribute to an ongoing debate of how an orderly binding of Aha1 and p23 cochaperones could exert thermodynamic control and succession in the ATPase cycle by suppressing the stochastically-driven conformational changes [53], [54], [70].

The recent mechanisms that related allosteric communications in proteins with frustration profiling have offered plausible, yet somewhat conflicting, models of a “frustration front” [130] and “rigidity path” [131]. The former model described allosteric coupling occurring via a front of highly frustrated residues moving in a “frustration tube”, where allosteric ligand binding would cause the tube residues to release tension and switch their conformational state [130]. The “rigidity path” model, that was based on a large scale analysis of allosteric proteins with the known active and inactive states, revealed the prevalence of rigid instantaneous coupling between the effector and catalytic sites found in ∼70% of the data set [131]. Subsequent discussions have noted that that the “rigidity path” and the “frustration front” may be complementary and potentially characterize distinct classes of proteins [115], [132]. According to our analysis, the topography of the allosterically connected network may be primarily determined by structurally rigid elements, including hinge points. Structural stability and a minimally frustrated nature of the hinge centers make them non-redundant and indispensable nodes similar to “discrete breathers” that correspond to the stiffest protein regions [115], [133], [134]. At the same time, binding partners, such as cochaperones or client proteins, can signal their presence by modulating the distribution and allocation of the locally frustrated residues in Hsp90 in order to temporarily deactivate or select specific communication pathways. Hence, structural and functional plasticity of Hsp90 that is modulated by cochaperones and utilized by client proteins requires a synergistic effort of both structurally rigid and mobile elements. In general, however, allosteric protein systems and macromolecular assemblies with different internal architecture and structural folds may use different mechanisms or their combinations in order to efficiently transmit signals between distant sites.

### Conclusions

In this work, an integrated computational modeling of allosteric regulation was employed to determine functionally important motifs that control dynamics and stability of the Hsp90-p23 and Hsp90-Aha1 interactions. According to our results, functional dynamics and allosteric interactions of Hsp90 can be selectively modulated by these co-chaperones. We have found that p23-mediated changes in the dynamic signature of Hsp90 are associated with stabilization of the Hsp90-N dimer interactions that are supported by the consolidation of the N-M contacts. These interactions may provide additional “molecular brakes” that could slow down an efficient transmission of the inter-domain allosteric signals. This distinctive signature of the chaperone dynamics in the presence of p23 is consistent with the experimental data and functional role of p23. Unlike p23, Aha1 can accelerate the progression of the Hsp90-ATPase cycle towards the dimerized closed form by directly “dialing” into the equilibrium motions of Hsp90 and promoting structural rearrangements that favor the closed chaperone form. The balance between structural rigidity and plasticity in Hsp90 may be associated with a dynamic coupling between minimally frustrated and locally frustrated residues, which can be modulated by cochaperones to regulate collective movements. These results have study suggested that the cochaperones Aha1 and p23 may modulate the ATPase activity by exerting spatial and temporal control over the collective motions of Hsp90 in order to accelerate or slow down conformational transitions. The protein structure network approach has provided a complementary outlook by quantifying the effects of cochaperones on conformational stability of the Hsp90 complexes. This analysis has identified dynamically stable networks of residues that can contribute to the strengthening of allosteric interactions. In summary, we have found that Aha1 and p23 could modulate the Hsp90-ATPase activity via specific targeting of the regulatory inter-domain regions that may control collective movements, participate in allosteric communications and be also responsible for structural stability of the Hsp90 complexes. Our results support a mechanism, according to which the global topology of the Hsp90 communication network may be determined by the architecture of the molecular chaperone and structural localization of the hinge sites, yet selection and activation of specific signal communication pathways could be cochaperone-dependent. These insights are consistent with a broad range of structural and biophysical experiments and could shed some light on how structural and dynamic changes induced by cochaperones could direct the ATPase cycle.

## Materials and Methods

### MD Simulations

MD refinement simulations of the low-energy docked models for the Hsp90-p23 and Hsp90-Aha1 complexes (each simulation was 5 ns duration) were carried out using NAMD 2.6 [135] with the CHARMM27 force field [136], [137] and the explicit TIP3P water model as implemented in NAMD 2.6. The employed MD protocol is consistent with the setup in MD simulations of the Hsp90 crystal structures [102] and was described in details in our earlier studies [102], [107], [138], [139]. In brief, the best docked conformations of yeast Hsp90 bound to ATP and p23 were used as starting points for MD refinement. Similarly to the setup described in [102], the original crystal structure of the of an Hsp90-ATP-p23/Sba1complex [38] was obtained by removal of the long disordered loop in the Hsp90-N domain, and the charged loop connecting the Hsp90-N and Hsp90-Mdomains, which is not present in the crystal structure, was modeled by a linker loop composed of 10 Gly residues with the aid of the ModLoop server [140]. All disordered loops in the Hsp90-M and Hsp90-C domain were modeled with ModLoop by preserving the original protein sequence [140]. The initial structures were solvated in a water box with the buffering distance of 10 Å. The system was subjected to initial minimization for 20,000 steps keeping protein backbone fixed which was followed by 20,000 steps of minimization without any constraints to allow system to relieve any steric contacts and relax freely. Equilibration was done in stages by gradually increasing the system temperature in steps of 20 K starting from 10 K until 310 K. At each stage, 30 ps equilibration was run using a restraint of 10 Kcalmol^−1^Å^−2^ on 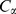 atoms. The system was then equilibrated for 300 ps at 310 K using Langevin piston (NPT) to achieve uniform pressure. After the restrains were removed the system was equilibrated for 300 ps to prepare the system for simulation. An NPT simulation was run on the equilibrated structure for 5 ns keeping the temperature at 310 K and pressure at 1 bar using Langevin piston coupling algorithm. Nonbonded van der Waals interactions were treated by using a switching function at 10Å and reaching zero at a distance of 12Å.

### The Gaussian Network Model

In the GNM representation [84]–[87], the protein structure is modeled as a network of *N* nodes identified by the 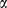-carbon atoms of proteins where the fluctuations of each node are isotropic and Gaussian. For nodes 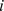 and 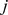, equilibrium position vectors, are 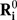 and 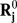, equilibrium distance vector is 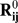, instantaneous fluctuation vectors are 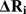 and 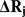, and instantaneous distance vector is 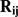. The difference between equilibrium position vector and instantaneous position vector of residue 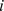 gives the instantaneous fluctuation vector 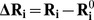. The instantaneous fluctuation vector between nodes 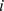 and 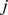 is expressed as 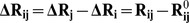. The topology of the protein structure is described by N × *N* Kirchhoff matrix of inter-residue contacts 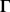, where the off-diagonal elements are −1 if the nodes are within a cutoff distance 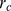 and zero otherwise. The diagonal elements represent the coordination number of each residue. For a network of N residues, the elements of the Kirchhoff matrix 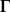 are defined as
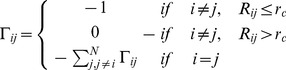
(1)


Bonded and nonbonded pairs of residues located within an interaction cutoff distance 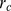 = 7.0 Å are assumed to be connected by springs with a uniform spring constant γ, which is the single parameter (force constant) of the Hookean potential [84]–[87]. The equilibrium dynamics of the structure results from the superposition of *N*-1 nonzero modes found by the eigenvalue decomposition of 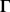. The elements of the *k*th eigenvector 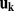 describe the displacements of the residues along the *k*th mode coordinate, and the *k*th eigenvalue, 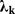, scales with the frequency of the *k*th mode, where 1≤ *k* ≤ *N* − 1. Assigning a uniform spring constant, 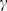 to all contacts, the cross-correlations between the fluctuations 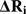 and 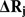 of residues (nodes) 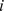 and 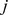 are computed. The GNM normal modes are found by diagonalization of the Kirchhoff matrix 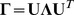. Here, 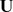 is a unitary matrix, 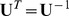 of the eigenvectors 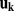 of 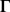 and 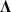 is the diagonal matrix of eigenvalues 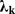. The frequency and shape of a mode is represented by its eigenvalue and eigenvector, respectively. 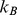 is the Boltzmann constant, 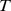 is the absolute temperature.
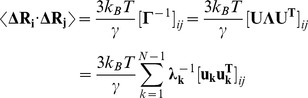
(2)


It follows that the contribution of an individual mode is expressed as
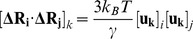
(3)


The mean square fluctuations of a given residue can be then evaluated as a sum over the contributions of all modes. The *i*th element of 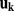 reflects the mobility of residue 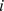 in the *k*th mode.
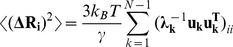
(4)


The slowest mode typically corresponds to the dominant mode of the functional global motions [84]–[87]. The GNM calculations of the MD-refined structures of the Hsp90 complexes were carried out using oGNM-based computations of equilibrium motions and conformational mobility profiles [86]. The normal mode analysis was performed using the WEBnm@ approach [141] and included computation and analysis of 20 low frequency modes. This approach utilized the approximate normal modes calculation method proven to be highly efficient and accurate for computations of low-frequency domain motions [142].

### Modeling of Allosteric Communication Pathways

The residue fluctuations in the GNM approach can determine the communication propensities between residues [81], [82]. A commute time between residues 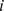 and 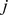, in terms of inverse of Kirchhoff matrix 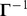 is given by the following expression:
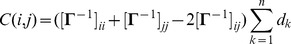
(5)


A commute time can be also presented in terms of instantaneous fluctuation vector between nodes 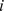 and 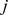 
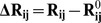


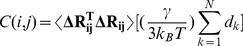
(6)



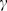
, 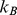 and 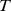 are the spring constant, the Boltzmann constant and the absolute temperature respectively as in the equations 2–4, and 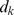 is the local interaction density at residue 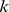. By definition, a diagonal element of the Kirchhoff matrix 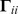 is equal to the degree of a node in GNM that represents the corresponding residue’s coordination number. This number is a measure of the local interaction density around a given residue. The mean square fluctuations play a dominant role in determining the communication propensities of a given pair of residues, namely the larger the mean square fluctuation the longer the commute time. In the CP analysis residues whose distances fluctuate with low intensity communicate with a higher efficiency than residues with larger fluctuations. Residues which display a high coordination number and local interaction density are often effective communicators according to the CP values.

### Protein-Protein Docking

The data-driven HADDOCK methodology [110] was used that for structural modeling and prediction of the Hsp90 complexes with the cochaperones p23 and Aha1. In this approach, structural and functional information is converted into AIR templates where the amino acids of interacting partners are designated as “active” and “passive” residues reflecting their role in binding. The detailed description and specification of the AIR sets for docking of the Hsp90-p23 and Hsp90-Aha1 complexes is presented in respective figure captions. The docking protocol consisted of three consecutive stages: (a) randomization of orientations followed by rigid body energy minimization (EM); (b) semi-flexible simulated annealing in torsion angle space, which consists of a rigid body MD search and first round of simulated annealing, followed by a second round semi-flexible simulated annealing during which side chains at the interface are free to move. A third round of semi-flexible simulated annealing is the next step of the search during which both side chains and backbone at the interface are free to move. A final refinement in Cartesian space with explicit solvent concludes the run [143]. Independent HADDOCK runs (∼5000) were conducted in docking simulations of the Hsp90-p23 complexes. A HADDOCK protocol that allows docking with conformational ensembles of multiple crystal structures of Hsp90 was adopted in simulations of the Hsp90-Aha1 complexes. The ensemble of Hsp90 conformations was extracted using previously reported all-atom MD simulations [107]. A 5000 independent HADDOCK runs was initially performed using only the crystal structure of the Aha1-N domain [68], [69]. The assembled sets of the AIR templates (Table S2) exploited the crystallographic information about the primary binding interface between the Aha1-N and the Hsp90-M domains [66]. In the course of each independent run, 2000 rigid-body structures were generated during the initial rigid body docking phase. A large number of independent runs (∼5000) combined with the default number of randomly generated initial structures (∼2000) in each of the conducted runs is expected to produce an adequate sampling of the conformational space for the Hsp90-cochaperone complexes. Docked solutions were judged by the total intermolecular energy:
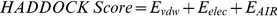
(7)where 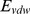 is the van der Waals energy, 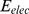 is the electrostatic energy, and 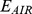 is the restraint contribution respectively. The best 200 docked models were submitted to cycles of the semi-flexible simulated annealing and final water refinement. The structures were ranked with standard weights [110], [143]. After the water refinement stage [143], the total energy was calculated as the weighted sum of the contributing terms:



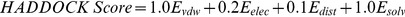
(8)

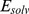
 is the solvation energy. The nonbonded intermolecular interactions were calculated with an 8.5 Å cutoff using the OPLS parameters. The dielectric constant epsilon was set to 10 in the vacuum part of the protocol and to 1 for the explicit solvent refinement. The secondary structure elements were kept intact during the simulated annealing refinement through hydrogen bond and dihedral angle restraints. The cutoff distance of 10 Å and a minimum cluster size of 5 structures were used in clustering of docked poses. The best scored structures from each cluster were reported and analyzed.

### Protein Structure Network Analysis

Protein structure network analysis is conducted using a web-tool that converts protein structures into graphs (http://vishgraph.mbu.iisc.ernet.in/GraProStr/). Protein structure networks are constructed by considering protein residues as nodes where edges between the nodes are constructed based on the noncovalent interactions as described in [93]–[100]. The pair of residues with the interaction strength 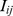 greater than a user-defined cut-off (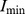) are connected by edges and produce a protein structure network graph for a given interaction strength 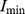. According to the analysis of a large number of protein structures, the optimal interaction strength 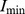 is typically in the range 2–4% [93], [97]. In our analysis, we considered any pair of residues to be connected if 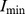 was greater than 3.0%. The protein network parameters such as clusters, hubs, cliques and communities were computed for cochaperone-free Hsp90 and Hsp90-cochaperone complexes. Protein hubs correspond to highly connected residues with more than three connections. A 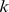-clique is defined as a set of 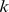 nodes that are represented by the protein residues in which each node is connected to all the other nodes. We have used a community definition from [93]–[98] according to which in a 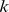-clique community two 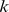-cliques share 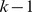 or 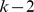 nodes. Protein cliques and communities are typically used to identify structurally stable protein regions [93]–[100]. In our analysis, we have averaged the stability of these parameters over the MD trajectories and analyzed the contribution of cliques and communities that were intact >75% of the simulation time. The protein ensemble for each system was constructed by considering ∼500 snapshots taken from previously reported all-atom MD simulations [107] of the crystal structure of yeast Hsp90 (PDB ID 2CG9) [38]; the crystal structures of the bacterial homologue HtpG in an open free form (PDB ID 2IOQ); an ADP-bound form (PDB ID 2IOP) [39], and from MD refinement trajectories of the Hsp90-p23 and Hsp90-Aha1 complexes.

### Local Frustration Analysis

The local frustration analysis adapted a recently proposed method of quantifying the degree of frustration manifested by spatially local protein interactions [126]–[129]. We employed the configurational frustration index that measures the relative stability of a particular native contact as a Z-score of the energy of the native pair with respect to a set of structural decoys generated by randomizing not only the residue identities but also the distance between the interacting amino acids [126], [127].The residue-based frustration index was computed via a web server (http://www.frustratometer.tk). This index measures the energetic stability of a particular native contact as compared to a set of all possible contacts sampled by automatic generation of ∼1000 appropriately distributed decoys for each contact. A contact was defined as minimally frustrated if its native energy was at the lower end of the distribution of decoy energies, and a frustration index as measured by a Z-score would be of 0.78 or higher magnitude. Conversely, a contact was defined as highly frustrated if its native energy was at the higher end of the distribution with a local frustration index lower than -1. If the native energy was in between these limits, the contact was defined as neutral.

## Supporting Information

Figure S1 Structural Analysis of the Aha1-N Docked Models. Structural analysis of the low energy docked complexes obtained with 5 different AIR sets (A–E). A sphere-based protein representation is used. The Hsp90 dimer is shown in grey, the Aha1-N domain is colored in green. (A) The first docked model was obtained with the active residues defined from the crystal structure of the Aha1-N bound with the yeast Hsp90-M domain [66]. The selected panel of active residues included the Hsp90-M residues L315, I388, V391, K387, K390, K394, K398 and the Aha1-N residues I64, L66, F100. The passive residues were defined as residues within 5Å of the actives. Additional active residues were taken from the NMR data [68] (excluding the Aha1-N to Hsp90-N interactions) and passives of these residues were defined as entire domains. (B) The second docked model was generated with the active residues determined from both the crystal structure and the NMR data (excluding the Aha1-N to Hsp90-N interactions), and all remaining residues were defined as passive. (C, D) The AIR sets used to generate these docked complexes defined the active residues only by the crystal structure [66] and passive residues either as entire domains (C) or as those within 5Å of the active residues (D). The last model (E) was obtained using the most detailed list of active residues derived from both crystal structures [66] and NMR mapping of the binding sites [68] including also the interactions between Aha1-N and Hsp90-N. All complexes obtained from the second iteration of HADDOCK refinement have experienced a partial loss of the secondary structure in the Aha1-N molecule. The best scoring models (A) and (D) resulted in the correct placement of the Aha1-N molecule and closely matched the NMR-based binding interface [68]. Residue L108 (shown in red spheres) was used as the “orientational landmark” for comparison with the NMR models.
(TIF)

Figure S2 The Scatter Graph Analysis of the Hsp90-Aha1 Docked Complexes. In the course of docking simulations with the “minimalist” AIR template that resulted in the best prediction, HADDOCK clustered 186 structures in 3 clusters which represented 93.0% of the water-refined docked models. The maximum number of models considered for clustering was 200. We illustrated the results of docking simulations by referring to the statistics of 3 clusters and scatter graphs between the HADDOCK score and the intermolecular interface RMSD (I-I RMSD) with respect to the lowest energy structure. The scatter graphs for top 3 clusters are based on water-refined models. The intermolecular interface I-I RMSD is based on residues forming the Hsp90-Aha1 interface. The top cluster 1 corresponds to the most populated cluster with the small standard deviation of HADDOCK scores sand the lowest average energy.
(TIF)

Table S1 The AIR Template for HADDOCK Simulations of the Hsp90-p23 Complex. The active residues used in HADDOCK modeling were defined as those involved in the Hsp90-p23 interactions according to [38]. Passive residues were defined as residues within a 5A radius of active residues.
(DOCX)

Table S2 The AIR Template for HADDOCK Simulations of the Hsp90-Aha1 Complex. The AIR sets used in HADDOCK to generate the predicted docked complexes defined the active residues only by the crystal structure of the Aha1-N bound with the yeast Hsp90-M domain [66]. Passive residues were defined as residues within a 5A radius of active residues. The selected panel of active residues included primarily the hydrophobic Hsp90-M residues L315, I388, V391 and the Aha1-N residues I64, L66, F100. The passive residues were defined as residues within 5Å of the actives.
(DOCX)

## References

[1] Pearl LH, Prodromou C (2001) Structure, function, and mechanism of the Hsp90 molecular chaperone. Adv Protein Chem 59: 157–186. 11868271 10.1016/s0065-3233(01)59005-1

[2] Richter K, Buchner J (2001) Hsp90: chaperoning signal transduction. J Cell Physiol 188: 281–290. 11473354 10.1002/jcp.1131

[3] Young JC, Moarefi I, Hartl FU (2001) Hsp90: a specialized but essential protein-folding tool. J Cell Biol 154: 267–273. 11470816 10.1083/jcb.200104079 PMC2150759

[4] Picard D (2002) Heat-shock protein 90, a chaperone for folding and regulation. Cell Mol Life Sci 59: 1640–1648. 12475174 10.1007/PL00012491 PMC11337538

[5] Young JC, Agashe VR, Siegers K, Hartl UF (2004) Pathways of chaperone-mediated protein folding in the cytosol. Nat Rev Mol Cell Biol 5: 781–791. 15459659 10.1038/nrm1492

[6] Zhao R, Davey M, Hsu YC, Kaplanek P, Tong A, et al (2005) Navigating the chaperone network: an integrative map of physical and genetic interactions mediated by the hsp90 chaperone. Cell 120: 715–727. 15766533 10.1016/j.cell.2004.12.024

[7] Pearl LH, Prodromou C (2006) Structure and mechanism of the Hsp90 molecular chaperone machinery. Annu Rev Biochem 75: 271–294. 16756493 10.1146/annurev.biochem.75.103004.142738

[8] Johnson JL (2012) Evolution and function of diverse Hsp90 homologs and cochaperone proteins. Biochim Biophys Acta 1823: 607–613. 22008467 10.1016/j.bbamcr.2011.09.020

[9] Prodromou C (2012) The ‘active life’ of Hsp90 complexes. Biochim Biophys Acta 1823: 614–623. 21840346 10.1016/j.bbamcr.2011.07.020 PMC3793855

[10] Li J, Soroka J, Buchner J (2012) The Hsp90 chaperone machinery: conformational dynamics and regulation by cochaperones. Biochim Biophys Acta 1823: 624–635. 21951723 10.1016/j.bbamcr.2011.09.003

[11] Echtenkamp FJ, Freeman BC (2012) Expanding the cellular molecular chaperone network through the ubiquitous cochaperones. Biochim Biophys Acta 1823(3): 668–673. 21889547 10.1016/j.bbamcr.2011.08.011

[12] Makhnevych T, Houry WA (2012) The role of Hsp90 in protein complex assembly. Biochim Biophys Acta 1823: 674–682. 21945180 10.1016/j.bbamcr.2011.09.001

[13] Theodoraki MA, Caplan AJ (2012) Quality control and fate determination of Hsp90 client proteins. Biochim Biophys Acta 1823: 683–688. 21871502 10.1016/j.bbamcr.2011.08.006 PMC3242914

[14] Queitsch C, Sangster TA, Lindquist S (2002) Hsp90 as a capacitor of phenotypic variation. Nature 417: 618–624. 12050657 10.1038/nature749

[15] Sangster TA, Lindquist S, Queitsch C (2004) Under cover: causes, effects and implications. Bioessays 26: 348–362. 15057933 10.1002/bies.20020

[16] Jarosz DF, Lindquist S (2010) Hsp90 and environmental stress transform the adaptive value of natural genetic variation. Science 330: 1820–1824. 21205668 10.1126/science.1195487 PMC3260023

[17] Röhl A, Rohrberg J, Buchner J (2013) The chaperone Hsp90: changing partners for demanding clients. Trends Biochem Sci 38: 253–262. 23507089 10.1016/j.tibs.2013.02.003

[18] Mayer MP, Nikolay R, Bukau B (2002) Aha, another regulator for hsp90 chaperones. Mol Cell 10: 1255–1256. 12503997 10.1016/s1097-2765(02)00793-1

[19] Richter K, Walter S, Buchner J (2004) The Co-chaperone Sba1 connects the ATPase reaction of Hsp90 to the progression of the chaperone cycle. J Mol Biol 342: 1403–1413. 15364569 10.1016/j.jmb.2004.07.064

[20] Weaver AJ, Sullivan WP, Felts SJ, Owen BA, Toft DO (2000) Crystal structure and activity of human p23, a heat shock protein 90 co-chaperone. J Biol Chem 275: 23045–23052. 10811660 10.1074/jbc.M003410200

[21] Morishima Y, Kanelakis KC, Murphy PJ, Lowe ER, Jenkins GJ, et al (2003) The hsp90 cochaperone p23 is the limiting component of the multiprotein hsp90/hsp70-based chaperone system in vivo where it acts to stabilize the client protein: hsp90 complex. J Biol Chem 278: 48754–48763. 14507910 10.1074/jbc.M309814200

[22] McLaughlin SH, Sobott F, Yao ZP, Zhang W, Nielsen PR, et al (2006) The co-chaperone p23 arrests the Hsp90 ATPase cycle to trap client proteins. J Mol Biol 356: 746–758. 16403413 10.1016/j.jmb.2005.11.085

[23] Forafonov F, Toogun OA, Grad I, Suslova E, Freeman BC, et al (2008) p23/Sba1p protects against Hsp90 inhibitors independently of its intrinsic chaperone activity. Mol Cell Biol 28: 3446–3456. 18362168 10.1128/MCB.02246-07 PMC2423160

[24] Richter K, Muschler P, Hainzl O, Reinstein J, Buchner J (2003) Sti1 is a non-competitive inhibitor of the Hsp90 ATPase. Binding prevents the N-terminal dimerization reaction during the atpase cycle. J Biol Chem 278: 10328–10333. 12525481 10.1074/jbc.M213094200

[25] Lee CT, Graf C, Mayer FJ, Richter SM, Mayer MP (2012) Dynamics of the regulation of Hsp90 by the co-chaperone Sti1. EMBO J 31: 1518–1528. 22354036 10.1038/emboj.2012.37 PMC3321184

[26] Schmid AB, Lagleder S, Gräwert MA, Röhl A, Hagn F, et al (2012) The architecture of functional modules in the Hsp90 co-chaperone Sti1/Hop. EMBO J 31: 1506–1517. 22227520 10.1038/emboj.2011.472 PMC3321170

[27] Zhang M, Botër M, Li K, Kadota Y, Panaretou B, et al (2008) Structural and functional coupling of Hsp90- and Sgt1-centred multi-protein complexes. EMBO J 27: 2789–2798. 18818696 10.1038/emboj.2008.190 PMC2556094

[28] Zhang M, Kadota Y, Prodromou C, Shirasu K, Pearl LH (2010) Structural basis for assembly of Hsp90-Sgt1-CHORD protein complexes: implications for chaperoning of NLR innate immunity receptors. Mol Cell 39: 269–281. 20670895 10.1016/j.molcel.2010.05.010 PMC2935968

[29] Pearl LH (2005) Hsp90 and Cdc37– a chaperone cancer conspiracy. Curr Opin Genet Dev 15: 55–61. 15661534 10.1016/j.gde.2004.12.011

[30] Mandal AK, Theodoraki MA, Nillegoda NB, Caplan AJ (2011) Role of molecular chaperones in biogenesis of the protein kinome. Methods Mol Biol 787: 75–81. 21898228 10.1007/978-1-61779-295-3_6

[31] McClellan AJ, Xia Y, Deutschbauer AM, Davis RW, Gerstein M, et al (2007) Diverse cellular functions of the Hsp90 molecular chaperone uncovered using systems approaches. Cell 131: 121–135. 17923092 10.1016/j.cell.2007.07.036

[32] Zhao R, Houry WA (2007) Molecular interaction network of the Hsp90 chaperone system. Adv Exp Med Biol 594: 27–36. 17205672 10.1007/978-0-387-39975-1_3

[33] Taipale M, Jarosz DF, Lindquist S (2010) Hsp90 at the hub of protein homeostasis: emerging mechanistic insights. Nat Rev Mol Cell Biol 11: 515–528. 20531426 10.1038/nrm2918

[34] Trepel J, Mollapour M, Giaccone G, Neckers L (2010) Targeting the dynamic HSP90 complex in cancer. Nat Rev Cancer 10: 537–549. 20651736 10.1038/nrc2887 PMC6778733

[35] Isaacs JS, Xu W, Neckers L (2003) Heat shock protein 90 as a molecular target for cancer therapeutics. Cancer Cell 3: 213–217. 12676580 10.1016/s1535-6108(03)00029-1

[36] Whitesell L, Lindquist SL (2005) Hsp90 and the chaperoning of cancer. Nat Rev Cancer 5: 761–772. 16175177 10.1038/nrc1716

[37] Xu W, Neckers L (2007) Targeting the molecular chaperone heat shock protein 90 provides a multifaceted effect on diverse cell signaling pathways of cancer cells. Clin Cancer Res 13: 1625–1629. 17363512 10.1158/1078-0432.CCR-06-2966

[38] Ali MM, Roe SM, Vaughan CK, Meyer P, Panaretou B, et al (2006) Crystal structure of an Hsp90-nucleotide-p23/Sba1 closed chaperone complex. Nature 440: 1013–1017. 16625188 10.1038/nature04716 PMC5703407

[39] Shiau AK, Harris SF, Southworth DR, Agard DA (2006) Structural Analysis of E. coli hsp90 reveals dramatic nucleotide-dependent conformational rearrangements. Cell 127: 329–340. 17055434 10.1016/j.cell.2006.09.027

[40] Dollins DE, Warren JJ, Immormino RM, Gewirth DT (2007) Structures of GRP94-nucleotide complexes reveal mechanistic differences between the hsp90 chaperones. Mol Cell 28: 41–56. 17936703 10.1016/j.molcel.2007.08.024 PMC2094010

[41] Vaughan CK, Gohlke U, Sobott F, Good VM, Ali MM, et al (2006) Structure of an Hsp90-Cdc37-Cdk4 complex. Mol. Cell 23: 697–707. 10.1016/j.molcel.2006.07.016 PMC5704897 16949366

[42] Krukenberg KA, Forster F, Rice LM, Sali A, Agard DA (2008) Multiple conformations of E. coli Hsp90 in solution: insights into the conformational dynamics of Hsp90. Structure 16: 755–765. 18462680 10.1016/j.str.2008.01.021 PMC2600884

[43] Graf C, Stankiewicz M, Kramer G, Mayer MP (2009) Spatially and kinetically resolved changes in the conformational dynamics of the Hsp90 chaperone machine. EMBO J 28: 602–613. 19165152 10.1038/emboj.2008.306 PMC2657576

[44] Ratzke C, Mickler M, Hellenkamp B, Buchner J, Hugel T (2010) Dynamics of heat shock protein 90 C-terminal dimerization is an important part of its conformational cycle. Proc Natl Acad Sci USA 107: 16101–16106. 20736353 10.1073/pnas.1000916107 PMC2941327

[45] Street TO, Lavery LA, Agard DA (2011) Substrate binding drives large-scale conformational changes in the Hsp90 molecular chaperone Mol Cell. 42: 96–105. 10.1016/j.molcel.2011.01.029 PMC3105473 21474071

[46] Southworth DR, Agard DA (2011) Client-loading conformation of the Hsp90 molecular chaperone revealed in the cryo-EM structure of the human Hsp90: Hop complex. Mol Cell 42: 771–781. 21700222 10.1016/j.molcel.2011.04.023 PMC3144320

[47] Cunningham CN, Southworth DR, Krukenberg KA, Agard DA (2012) The conserved arginine 380 of Hsp90 is not a catalytic residue, but stabilizes the closed conformation required for ATP hydrolysis. Protein Sci 21: 1162–1171. 22653663 10.1002/pro.2103 PMC3537237

[48] Street TO, Lavery LA, Verba KA, Lee CT, Mayer MP, et al (2012) Cross-monomer substrate contacts reposition the Hsp90 N-terminal domain and prime the chaperone activity. J Mol Biol 415: 3–15. 22063096 10.1016/j.jmb.2011.10.038 PMC3282117

[49] Genest O, Reidy M, Street TO, Hoskins JR, Camberg JL, et al (2013) Uncovering a region of heat shock protein 90 important for client binding in E. coli and chaperone function in yeast. Mol Cell 49: 464–473. 23260660 10.1016/j.molcel.2012.11.017 PMC3570620

[50] Pearl LH, Prodromou C, Workman P (2008) The Hsp90 molecular chaperone: an open and shut case for treatment. Biochem J 410: 439–453. 18290764 10.1042/BJ20071640

[51] Krukenberg KA, Street TO, Lavery LA, Agard DA (2011) Conformational dynamics of the molecular chaperone Hsp90. Q Rev Biophys 44: 229–255. 21414251 10.1017/S0033583510000314 PMC5070531

[52] Jackson SE (2013) Hsp90: structure and function. Top Curr Chem 328: 155–240. 22955504 10.1007/128_2012_356

[53] Mickler M, Hessling M, Ratzke C, Buchner J, Hugel T (2009) The large conformational changes of Hsp90 are only weakly coupled to ATP hydrolysis. Nat Struct Mol Biol 16: 281–286. 19234469 10.1038/nsmb.1557

[54] Ratzke C, Nguyen MN, Mayer MP, Hugel T (2012) From a ratchet mechanism to random fluctuations evolution of Hsp90’s mechanochemical cycle. J Mol Biol 423: 462–471. 22878379 10.1016/j.jmb.2012.07.026

[55] Martinez-Yamout MA, Venkitakrishnan RP, Preece NE, Kroon G, Wright PE, et al (2006) Localization of sites of interaction between p23 and Hsp90 in solution. J Biol Chem 281: 14457–1464. 16565516 10.1074/jbc.M601759200

[56] Karagöz GE, Duarte AM, Ippel H, Uetrecht C, Sinnige T, et al (2011) N-terminal domain of human Hsp90 triggers binding to the cochaperone p23. Proc Natl Acad Sci USA 108: 580–555. 21183720 10.1073/pnas.1011867108 PMC3021017

[57] Didenko T, Duarte AM, Karagöz GE, Rüdiger SG (2012) Hsp90 structure and function studied by NMR spectroscopy. Biochim Biophys Acta. 1823: 636–647. 10.1016/j.bbamcr.2011.11.009 22155720

[58] Panaretou B, Siligardi G, Meyer P, Maloney A, Sullivan JK, et al (2002) Activation of the ATPase activity of hsp90 by the stress-regulated cochaperone aha1. Mol Cell 10: 1307–1318. 12504007 10.1016/s1097-2765(02)00785-2

[59] Lotz GP, Lin H, Harst A, Obermann WM (2003) Aha1 binds to the middle domain of Hsp90, contributes to client protein activation, and stimulates the ATPase activity of the molecular chaperone. J Biol Chem 278: 17228–17235. 12604615 10.1074/jbc.M212761200

[60] Hessling M, Richter K, Buchner J (2009) Dissection of the ATP-induced conformational cycle of the molecular chaperone Hsp90. Nat Struct Mol Biol 16: 287–293. 19234467 10.1038/nsmb.1565

[61] Meyer P, Prodromou C, Hu B, Vaughan C, Roe SM, et al (2003) Structural and functional analysis of the middle segment of hsp90: implications for ATP hydrolysis and client protein and cochaperone interactions. Mol Cell 11: 647–658. 12667448 10.1016/s1097-2765(03)00065-0

[62] Harst A, Lin H, Obermann WM (2005) Aha1 competes with Hop, p50 and p23 for binding to the molecular chaperone Hsp90 and contributes to kinase and hormone receptor activation. Biochem J 387: 789–796. 15584899 10.1042/BJ20041283 PMC1135010

[63] Siligardi G, Hu B, Panaretou B, Piper PW, Pearl LH, et al (2004) Co-chaperone regulation of conformational switching in the Hsp90 ATPase cycle. J Biol Chem 279: 51989–51998. 15466438 10.1074/jbc.M410562200

[64] Gaiser AM, Kretzschmar A, Richter K (2010) Cdc37-Hsp90 complexes are responsive to nucleotide-induced conformational changes and binding of further cofactors. J Biol Chem 285: 40921–40932. 20880838 10.1074/jbc.M110.131086 PMC3003392

[65] Sun L, Prince T, Manjarrez JR, Scroggins BT, Matts RL (2012) Characterization of the interaction of Aha1 with components of the Hsp90 chaperone machine and client proteins. Biochim Biophys Acta 1823: 1092–1101. 22504172 10.1016/j.bbamcr.2012.03.014

[66] Meyer P, Prodromou C, Liao C, Hu B, Roe SM, et al (2004) Structural basis for recruitment of the ATPase activator Aha1 to the Hsp90 chaperone machinery. EMBO J. 23: 1402–1410. 10.1038/sj.emboj.7600141 PMC381413 15039704

[67] Meyer P, Prodromou C, Liao C, Hu B, Mark Roe S, et al (2004) Structural basis for recruitment of the ATPase activator Aha1 to the Hsp90 chaperone machinery. EMBO J. 23: 511–519. 10.1038/sj.emboj.7600060 PMC1271799 14739935

[68] Retzlaff M, Hagn F, Mitschke L, Hessling M, Gugel F, et al (2010) Asymmetric activation of the hsp90 dimer by its cochaperone aha1. Mol Cell 37: 344–354. 20159554 10.1016/j.molcel.2010.01.006

[69] Koulov AV, LaPointe P, Lu B, Razvi A, Coppinger J, et al (2010) Biological and structural basis for Aha1 regulation of Hsp90 ATPase activity in maintaining proteostasis in the human disease cystic fibrosis. Mol Biol Cell 21: 871–884. 20089831 10.1091/mbc.E09-12-1017 PMC2836968

[70] Li J, Richter K, Reinstein J, Buchner J (2013) Integration of the accelerator Aha1 in the Hsp90 cochaperone cycle. Nat Struct Mol Biol 20: 326–331. 23396352 10.1038/nsmb.2502

[71] Hartson SD, Matts RL (2011) Approaches for defining the Hsp90-dependent proteome. Biochim Biophys Acta 1823: 656–667. 21906632 10.1016/j.bbamcr.2011.08.013 PMC3276727

[72] Tsaytler PA, Krijgsveld J, Goerdayal SS, Rüdiger S, Egmond MR (2009) Novel Hsp90 partners discovered using complementary proteomic approaches. Cell Stress Chaperones 14: 629–638. 19396626 10.1007/s12192-009-0115-z PMC2866955

[73] Taipale M, Krykbaeva I, Koeva M, Kayatekin C, Westover KD, et al (2012) Quantitative analysis of HSP90-client interactions reveals principles of substrate recognition. Cell 150: 987–1001. 22939624 10.1016/j.cell.2012.06.047 PMC3894786

[74] Sharma K, Vabulas RM, Macek B, Pinkert S, Cox J, et al (2012) Quantitative proteomics reveals that Hsp90 inhibition preferentially targets kinases and the DNA damage response. Mol Cell Proteomics 11: M111.014654. 10.1074/mcp.M111.014654 PMC3316734 22167270

[75] Wu Z, Moghaddas Gholami A, Kuster B (2012) Systematic identification of the HSP90 candidate regulated proteome. Mol Cell Proteomics 11: M111.016675. 10.1074/mcp.M111.016675 PMC3433914 22337586

[76] Haupt A, Joberty G, Bantscheff M, Fröhlich H, Stehr H, et al (2012) Hsp90 inhibition differentially destabilises MAP kinase and TGF-beta signalling components in cancer cells revealed by kinase-targeted chemoproteomics. BMC Cancer 12: 38. 22277058 10.1186/1471-2407-12-38 PMC3342885

[77] Cui Q, Karplus M (2008) Allostery and cooperativity revisited. Protein Sci 17: 1295–1307. 18560010 10.1110/ps.03259908 PMC2492820

[78] Goodey NM, Benkovic SJ (2008) Allosteric regulation and catalysis emerge via a common route. Nat Chem Biol 4: 474–482. 18641628 10.1038/nchembio.98

[79] Tsai CJ, Sol AD, Nussinov R (2009) Protein allostery, signal transmission and dynamics: a classification scheme of allosteric mechanisms. Mol Biosyst 5: 207–216. 19225609 10.1039/b819720b PMC2898650

[80] Sol AD, Sol, Tsai CJ, Ma B, Nussinov R (2009) The origin of allosteric functional modulation: multiple pre-existing pathways. Structure 17: 1042–1050. 19679084 10.1016/j.str.2009.06.008 PMC2749652

[81] Chennubhotla C, Bahar I (2007) Signal propagation in proteins and relation to equilibrium fluctuations. PLoS Comput Biol 3: 1716–1726. 17892319 10.1371/journal.pcbi.0030172 PMC1988854

[82] Bahar I, Chennubhotla C, Tobi D (2007) Intrinsic dynamics of enzymes in the unbound state and relation to allosteric regulation. Curr Opin Struct Biol 17: 633–640. 18024008 10.1016/j.sbi.2007.09.011 PMC2197162

[83] Daily MD, Gray JJ (2009) Allosteric communication occurs via networks of tertiary and quaternary motions in proteins. PLoS Comput Biol 5: e1000293. 19229311 10.1371/journal.pcbi.1000293 PMC2634971

[84] Bahar I, Lezon TR, Yang LW, Eyal E (2010) Global dynamics of proteins: bridging between structure and function. Annu Rev Biophys 39: 23–42. 20192781 10.1146/annurev.biophys.093008.131258 PMC2938190

[85] Haliloglu T, Bahar I, Erman B (1997) Gaussian dynamics of folded proteins. Phys Rev Lett 79: 3090–3093.

[86] Yang LW, Rader AJ, Liu X, Jursa CJ, Chen SC, et al.. (2006) oGNM: online computation of structural dynamics using the Gaussian Network Model. Nucleic Acids Res 34(Web Server issue): W24–W31. 10.1093/nar/gkl084 PMC1538811 16845002

[87] Eyal E, Yang LW, Bahar I (2006) Anisotropic network model: systematic evaluation and a new web interface. Bioinformatics 22: 2619–2627. 16928735 10.1093/bioinformatics/btl448

[88] Ma J (2005) Usefulness and limitations of normal mode analysis in modeling dynamics of biomolecular complexes. Structure 13: 373–380. 15766538 10.1016/j.str.2005.02.002

[89] Bahar I, Rader AJ (2005) Coarse-grained normal mode analysis in structural biology. Curr Opin Struc Biol 15: 1–7. 10.1016/j.sbi.2005.08.007 PMC1482533 16143512

[90] Popovych N, Sun S, Ebright RH, Kalodimos CG (2006) Dynamically driven protein allostery. Nat Struct Mol Biol 13: 831–838. 16906160 10.1038/nsmb1132 PMC2757644

[91] Hardy JA, Wells JA (2004) Searching for new allosteric sites in enzymes. Curr Opin Struct Biol 14: 706–715. 15582395 10.1016/j.sbi.2004.10.009

[92] Keskin O, Jernigan RL, Bahar I (2000) Proteins with similar architecture exhibit similar large-scale dynamic behavior. Biophys J 78: 2093–2106. 10733987 10.1016/S0006-3495(00)76756-7 PMC1300801

[93] Brinda KV, Vishveshwara S (2005) A network representation of protein structures: implications for protein stability. Biophys J 89: 4159–4170. 16150969 10.1529/biophysj.105.064485 PMC1366981

[94] Ghosh A, Vishveshwara S (2007) A study of communication pathways in methionyl-tRNA synthetase by molecular dynamics simulations and structure network analysis. Proc Natl Acad Sci USA 104: 15711–15716. 17898174 10.1073/pnas.0704459104 PMC2000407

[95] Ghosh A, Vishveshwara S (2008) Variations in clique and community patterns in protein structures during allosteric communication: investigation of dynamically equilibrated structures of methionyl tRNA synthetase complexes. Biochemistry 47: 11398–11407. 18842003 10.1021/bi8007559

[96] Bhattacharyya M, Ghosh A, Hansia P, Vishveshwara S (2010) Allostery and conformational free energy changes in human tryptophanyl-tRNA synthetase from essential dynamics and structure networks. Proteins 78: 506–517. 19768679 10.1002/prot.22573

[97] Vijayabaskar MS, Vishveshwara S (2010) Interaction energy based protein structure networks. Biophys J 99: 3704–3715. 21112295 10.1016/j.bpj.2010.08.079 PMC2998601

[98] Bhattacharyya M, Vishveshwara S (2010) Elucidation of the conformational free energy landscape in H.pylori LuxS and its implications to catalysis. BMC Struct Biol 10: 27. 20704697 10.1186/1472-6807-10-27 PMC2929236

[99] Sukhwal A, Bhattacharyya M, Vishveshwara S (2011) Network approach for capturing ligand-induced subtle global changes in protein structures. Acta Crystallogr D Biol Crystallogr 67: 429–439. 21543845 10.1107/S0907444911007062

[100] Bhattacharyya M, Vishveshwara S (2011) Probing the allosteric mechanism in pyrrolysyl-tRNA synthetase using energy-weighted network formalism. Biochemistry 50: 6225–6236. 21650159 10.1021/bi200306u

[101] Colombo G, Morra G, Meli M, Verkhivker G (2008) Understanding ligand-based modulation of the Hsp90 molecular chaperone dynamics at atomic resolution. Proc Natl Acad Sci USA 105: 7976–7981. 18511558 10.1073/pnas.0802879105 PMC2402385

[102] Morra G, Verkhivker G (2009) Colombo (2009) Modeling signal propagation mechanisms and ligand-based conformational dynamics of the Hsp90 molecular chaperone full length dimer. PLoS Comput Biol 5: e1000323. 19300478 10.1371/journal.pcbi.1000323 PMC2649446

[103] Verkhivker GM, Dixit A, Morra G, Colombo G (2009) Structural and computational biology of the molecular chaperone Hsp90: from understanding molecular mechanisms to computer-based inhibitor design. Curr Top Med Chem 9: 1369–1385. 19860735 10.2174/156802609789895700

[104] Morra G, Neves MAC, Plescia CJ, Tsutsumi S, Neckers L, et al (2010) Dynamics-based discovery of allosteric inhibitors: Selection of new ligands for the C-terminal domain of Hsp90. J Chem Theory Comput 6: 2978–2989. 26616092 10.1021/ct100334n PMC7575213

[105] Matts RL, Brandt GE, Lu Y, Dixit A, Mollapour M, et al (2011) A systematic protocol for the characterization of Hsp90 modulators. Bioorg Med Chem 19: 684–692. 21129982 10.1016/j.bmc.2010.10.029 PMC4618652

[106] Matts RL, Dixit A, Peterson LB, Sun L, Voruganti S, et al (2011) Elucidation of the Hsp90 C-terminal inhibitor binding site. ACS Chem Biol 6: 800–807. 21548602 10.1021/cb200052x PMC3164513

[107] Dixit A, Verkhivker GM (2012) Probing molecular mechanisms of the Hsp90 chaperone: Biophysical modeling identifies key regulators of functional dynamics. PLoS One 7: e37605. 22624053 10.1371/journal.pone.0037605 PMC3356286

[108] Morra G, Potestio R, Micheletti C, Colombo G (2012) Corresponding functional dynamics across the Hsp90 Chaperone family: insights from a multiscale analysis of MD simulations. PLoS Comput Biol 8: e1002433. 22457611 10.1371/journal.pcbi.1002433 PMC3310708

[109] Seifert C, Gräter F (2012) Force distribution reveals signal transduction in E. coli Hsp90. Biophys J 103: 2195–2202. 23200053 10.1016/j.bpj.2012.09.008 PMC3512052

[110] Dominguez C, Boelens R, Bonvin AM (2003) HADDOCK: a protein-protein docking approach based on biochemical or biophysical information. J Am Chem Soc 125: 1731–1737. 12580598 10.1021/ja026939x

[111] Lee CC, Lin TW, Ko TP, Wang AH (2011) The hexameric structures of human heat shock protein 90. PLoS One 6: e19961. 21647436 10.1371/journal.pone.0019961 PMC3102065

[112] Chen B, Zhong D, Monteiro A (2006) Comparative genomics and evolution of the HSP90 family of genes across all kingdoms of organisms. BMC Genomics 7: 156. 16780600 10.1186/1471-2164-7-156 PMC1525184

[113] Retzlaff M, Stahl M, Eberl HC, Lagleder S, Beck J, et al (2009) Hsp90 is regulated by a switch point in the C-terminal domain. EMBO Rep 10: 1147–1153. 19696785 10.1038/embor.2009.153 PMC2759728

[114] Csermely P (2008) Creative elements: network-based predictions of active centers in proteins, cellular and social networks. Trends Biochem Sci 33: 569–576. 18945619 10.1016/j.tibs.2008.09.006

[115] Csermely P, Palotai R, Nussinov R (2010) Induced fit, conformational selection and independent dynamic segments: an extended view of binding events. Trends Biochem Sci 35: 539–546. 20541943 10.1016/j.tibs.2010.04.009 PMC3018770

[116] Millson SH, Truman AW, Wolfram F, King V, Panaretou B, et al (2004) Investigating the protein-protein interactions of the yeast Hsp90 chaperone system by two-hybrid analysis: potential uses and limitations of this approach Cell Stress Chaperones. 9: 359–368. 10.1379/CSC-29R1.1 PMC1065275 15633294

[117] Mollapour M, Tsutsumi S, Donnelly AC, Beebe K, Tokita MJ, et al (2010) Swe1Wee1-dependent tyrosine phosphorylation of Hsp90 regulates distinct facets of chaperone function. Mol Cell 37: 333–343. 20159553 10.1016/j.molcel.2010.01.005 PMC2824606

[118] Mollapour M, Tsutsumi S, Truman AW, Xu W, Vaughan CK, et al (2011) Threonine 22 phosphorylation attenuates Hsp90 interaction with cochaperones and affects its chaperone activity. Mol Cell 41: 672–681. 21419342 10.1016/j.molcel.2011.02.011 PMC3062913

[119] Zurawska A, Urbanski J, Matuliene J, Baraniak J, Klejman MP, et al (2010) Mutations that increase both Hsp90 ATPase activity in vitro and Hsp90 drug resistance in vivo. Biochim Biophys Acta 1803: 575–583. 20226818 10.1016/j.bbamcr.2010.03.002

[120] Cunningham CN, Krukenberg KA, Agard DA (2008) Intra- and intermonomer interactions are required to synergistically facilitate ATP hydrolysis in Hsp90. J Biol Chem 283: 21170–21178. 18492664 10.1074/jbc.M800046200 PMC2475720

[121] Xu W, Mollapour M, Prodromou C, Wang S, Scroggins BT, et al (2012) Dynamic tyrosine phosphorylation modulates cycling of the HSP90-P50(CDC37)-AHA1 chaperone machine. Mol Cell 47: 434–443. 22727666 10.1016/j.molcel.2012.05.015 PMC3418412

[122] Soroka J, Wandinger SK, Mäusbacher N, Schreiber T, Richter K, et al (2012) Conformational switching of the molecular chaperone Hsp90 via regulated phosphorylation. Mol Cell 45: 517–528. 22365831 10.1016/j.molcel.2011.12.031

[123] Miyashita O, Onuchic JN, Wolynes PG (2003) Nonlinear elasticity, protein quakes, and the energy landscapes of functional transitions in proteins. Proc Natl Acad Sci USA 100: 12570–12575. 14566052 10.1073/pnas.2135471100 PMC240658

[124] Okazaki KI, Koga N, Takada S, Onuchic JN, Wolynes PG (2006) Multiple-basin energy landscapes for large-amplitude conformational motions of proteins: Structure-based molecular dynamics simulations. Proc Natl Acad Sci USA 103: 11844–11849. 16877541 10.1073/pnas.0604375103 PMC1567665

[125] Okazaki KI, Takada S (2008) Dynamic energy landscape view of coupled binding and protein conformational change: induced-fit versus population-shift mechanisms. Proc Natl Acad Sci USA 105: 11182–11187. 18678900 10.1073/pnas.0802524105 PMC2516237

[126] Ferreiro DU, Hegler JA, Komives EA, Wolynes PG (2007) Localizing frustration in native proteins and protein assemblies Proc Natl Acad Sci USA. 104: 19819–19824. 10.1073/pnas.0709915104 PMC2148382 18077414

[127] Sutto L, Lätzer J, Hegler JA, Ferreiro DU, Wolynes PG (2007) Consequences of localized frustration for the folding mechanism of the IM7 protein. Proc Natl Acad Sci USA 104 104: 19825–19830. 10.1073/pnas.0709922104 PMC2148258 18077415

[128] Li W, Wolynes PG, Takada S (2011) Frustration, specific sequence dependence, and nonlinearity in large-amplitude fluctuations of allosteric proteins. Proc Natl Acad Sci USA 108: 3504–3509. 21307307 10.1073/pnas.1018983108 PMC3048140

[129] Ferreiro DU, Hegler JA, Komives EA, Wolynes PG (2011) On the role of frustration in the energy landscapes of allosteric proteins. Proc Natl Acad Sci USA 108: 3499–3503. 21273505 10.1073/pnas.1018980108 PMC3048099

[130] Zhuravlev PI, Papoian GA (2010) Protein functional landscapes, dynamics, allostery: a tortuous path towards a universal theoretical framework. Q Rev Biophys 43: 295–332. 20819242 10.1017/S0033583510000119

[131] Rader AJ, Brown SM (2011) Correlating allostery with rigidity. Mol Biosyst 7: 464–471. 21060909 10.1039/c0mb00054j

[132] Csermely P, Sandhu KS, Hazai E, Hoksza Z, Kiss HJ, et al (2012) Disordered proteins and network disorder in network descriptions of protein structure, dynamics and function: hypotheses and a comprehensive review. Curr Protein Pept Sci 13: 19–33. 22044146 10.2174/138920312799277992

[133] Piazza F, Sanejouand YH (2008) Discrete breathers in protein structures. Phys Biol 5: 026001. 18451466 10.1088/1478-3975/5/2/026001

[134] Luccioli S, Imparato A, Lepri S, Piazza F, Torcini A (2011) Discrete breathers in a realistic coarse-grained model of proteins. Phys Biol 8: 046008. 21670494 10.1088/1478-3975/8/4/046008

[135] Phillips JC, Braun R, Wang W, Gumbart J, Tajkhorshid E, et al (2005) Scalable molecular dynamics with NAMD. J Comput Chem 26: 1781–1802. 16222654 10.1002/jcc.20289 PMC2486339

[136] MacKerell AD Jr, Bashford D, Bellott M, Dunbrack RL Jr, Evanseck JD, et al (1998) All-atom empirical potential for molecular modeling and dynamics studies of proteins. J Phys Chem B 102: 3586–3616. 24889800 10.1021/jp973084f

[137] MacKerell AD Jr, Banavali N, Foloppe N (2001) Development and current status of the CHARMM force field for nucleic acids. Biopolymers 56: 257–265. 10.1002/1097-0282(2000)56:4<257::AID-BIP10029>3.0.CO;2-W 11754339

[138] Dixit A, Verkhivker G (2009) Hierarchical modeling of activation mechanisms in the ABL and EGFR kinase domains: thermodynamic and mechanistic catalysts of kinase activation by cancer mutations PLoS Comput Biol. 5: e1000487. 10.1371/journal.pcbi.1000487 PMC2722018 19714203

[139] Dixit A, Verkhivker G (2011) Computational modeling of allosteric communication reveals organizing principles of mutation-induced signaling in ABL and EGFR kinases. PLoS Comput Biol 7: e1002179. 21998569 10.1371/journal.pcbi.1002179 PMC3188506

[140] Fiser A, Do RK, Sali A (2000) Modeling of loops in protein structures. Prot Sci 9: 1753–1773. 10.1110/ps.9.9.1753 PMC2144714 11045621

[141] Hollup SM, Salensminde G, Reuter N (2005) WEBnm: a web application for normal mode analyses of proteins. BMC Bioinformatics. 6: 52. 10.1186/1471-2105-6-52 PMC1274249 15762993

[142] Hinsen K (1998) Analysis of domain motions by approximate normal mode calculations. Proteins 33: 417–429. 9829700 10.1002/(sici)1097-0134(19981115)33:3<417::aid-prot10>3.0.co;2-8

[143] van Dijk AD, Bonvin AM (2006) Solvated docking: introducing water into the modeling of biomolecular complexes. Bioinformatics 22: 2340–2347. 16899489 10.1093/bioinformatics/btl395

