# Supplementary figures and images for: Differential Modulation of Functional Dynamics and Allosteric Interactions in the Hsp90-Cochaperone Complexes with p23 and Aha1: A Computational Study
Source: PLoS One. 2013 Aug 19;8(8):e71936. doi: 10.1371/journal.pone.0071936 (PMC3747073; doi:10.1371/journal.pone.0071936)

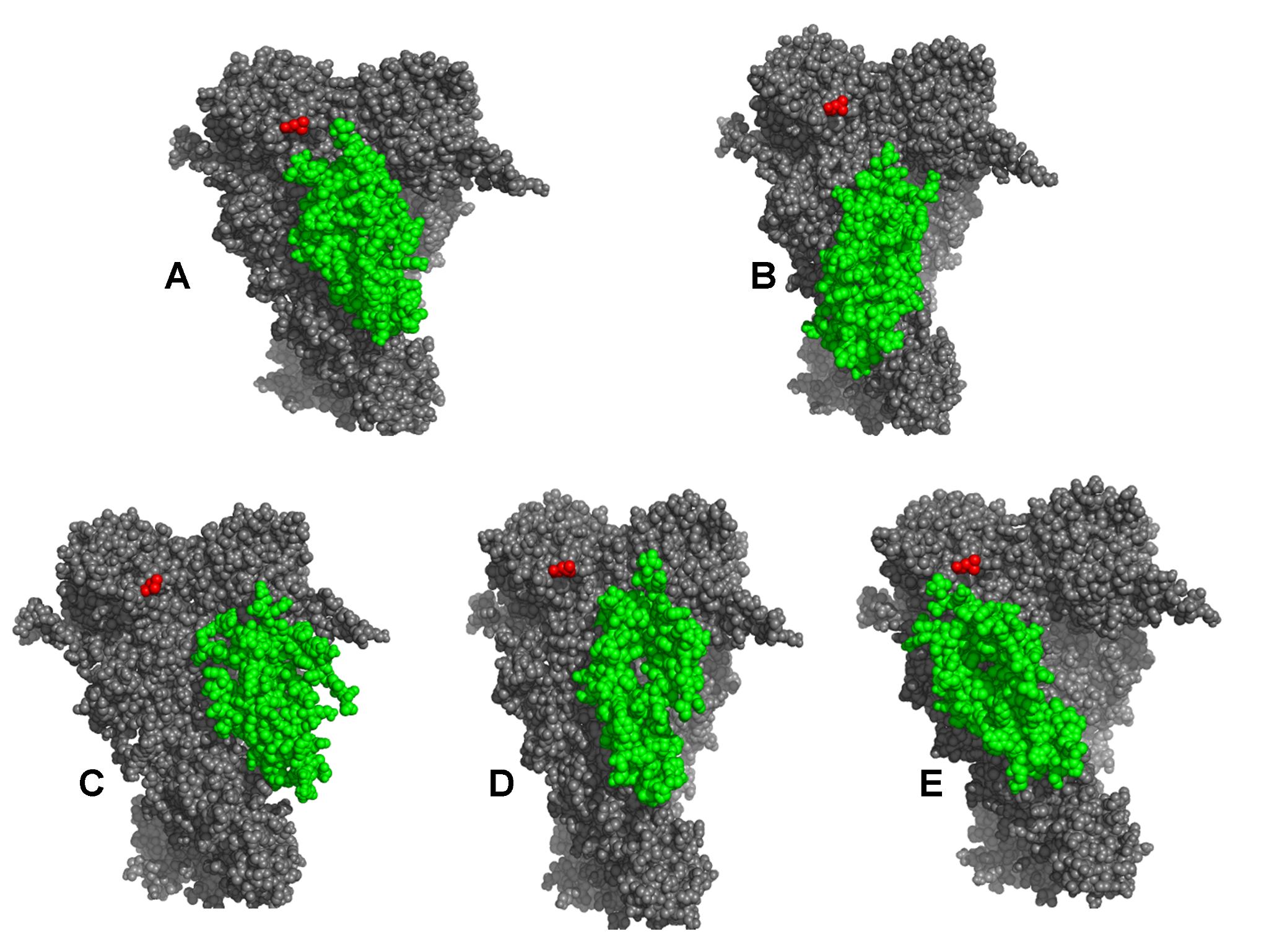

Supplement: Figure S1 — Structural Analysis of the Aha1-N Docked Models. Structural analysis of the low energy docked complexes obtained with 5 different AIR sets (A–E). A sphere-based protein representation is used. The Hsp90 dimer is shown in grey, the Aha1-N domain is colored in green. (A) The first docked model was obtained with the active residues defined from the crystal structure of the Aha1-N bound with the yeast Hsp90-M domain [66]. The selected panel of active residues included the Hsp90-M residues L315, I388, V391, K387, K390, K394, K398 and the Aha1-N residues I64, L66, F100. The passive residues were defined as residues within 5Å of the actives. Additional active residues were taken from the NMR data [68] (excluding the Aha1-N to Hsp90-N interactions) and passives of these residues were defined as entire domains. (B) The second docked model was generated with the active residues determined from both the crystal structure and the NMR data (excluding the Aha1-N to Hsp90-N interactions), and all remaining residues were defined as passive. (C, D) The AIR sets used to generate these docked complexes defined the active residues only by the crystal structure [66] and passive residues either as entire domains (C) or as those within 5Å of the active residues (D). The last model (E) was obtained using the most detailed list of active residues derived from both crystal structures [66] and NMR mapping of the binding sites [68] including also the interactions between Aha1-N and Hsp90-N. All complexes obtained from the second iteration of HADDOCK refinement have experienced a partial loss of the secondary structure in the Aha1-N molecule. The best scoring models (A) and (D) resulted in the correct placement of the Aha1-N molecule and closely matched the NMR-based binding interface [68]. Residue L108 (shown in red spheres) was used as the “orientational landmark” for comparison with the NMR models. (TIF) [file pone.0071936.s001.tif]

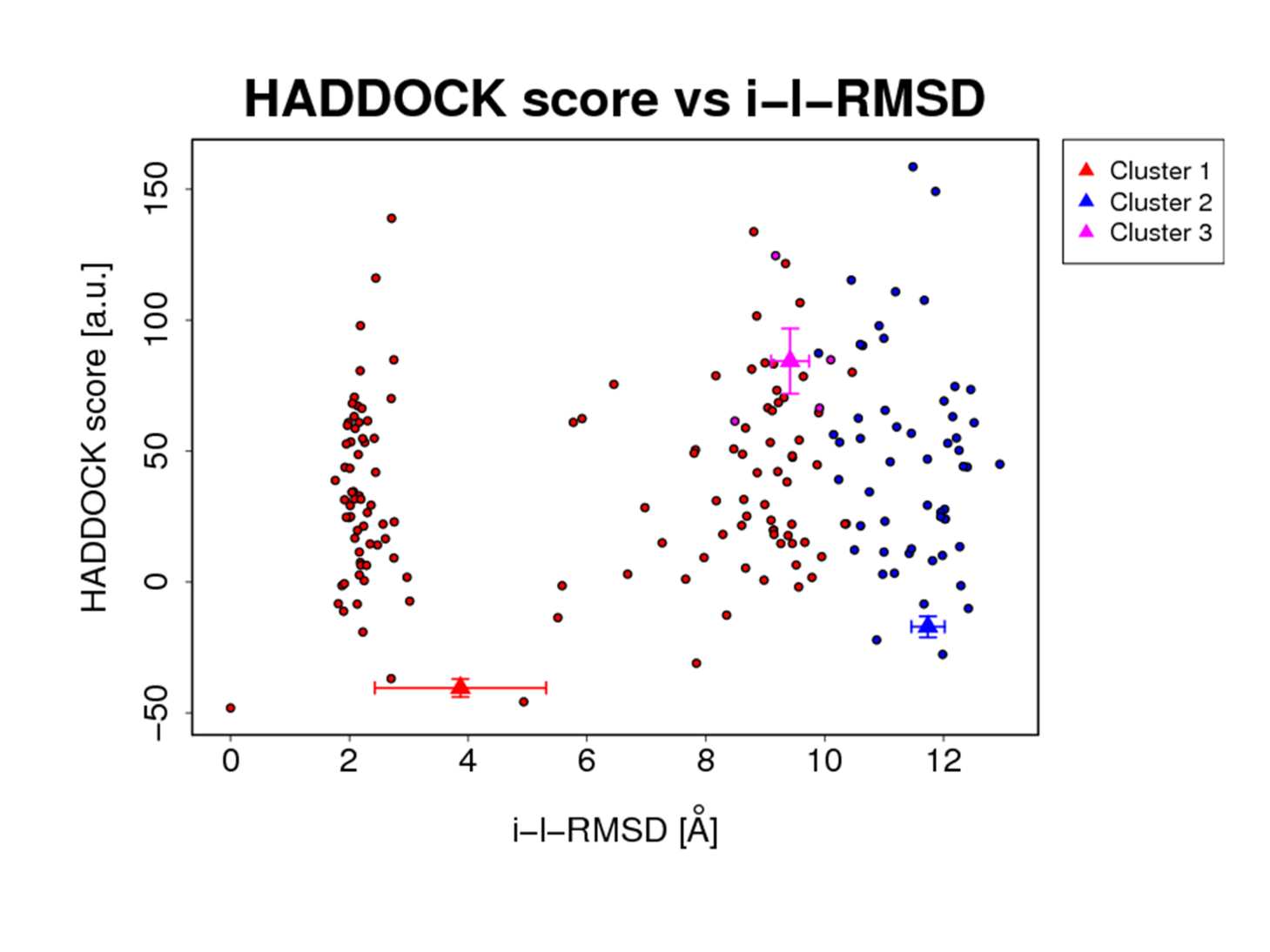

Supplement: Figure S2 — The Scatter Graph Analysis of the Hsp90-Aha1 Docked Complexes. In the course of docking simulations with the “minimalist” AIR template that resulted in the best prediction, HADDOCK clustered 186 structures in 3 clusters which represented 93.0% of the water-refined docked models. The maximum number of models considered for clustering was 200. We illustrated the results of docking simulations by referring to the statistics of 3 clusters and scatter graphs between the HADDOCK score and the intermolecular interface RMSD (I-I RMSD) with respect to the lowest energy structure. The scatter graphs for top 3 clusters are based on water-refined models. The intermolecular interface I-I RMSD is based on residues forming the Hsp90-Aha1 interface. The top cluster 1 corresponds to the most populated cluster with the small standard deviation of HADDOCK scores sand the lowest average energy. (TIF) [file pone.0071936.s002.tif]
