# Supplementary material for: Differential Modulation of Functional Dynamics and Allosteric Interactions in the Hsp90-Cochaperone Complexes with p23 and Aha1: A Computational Study
Source: PLoS One. 2013 Aug 19;8(8):e71936. doi: 10.1371/journal.pone.0071936 (PMC3747073; doi:10.1371/journal.pone.0071936)
Supplement: Table S1 — The AIR Template for HADDOCK Simulations of the Hsp90-p23 Complex. The active residues used in HADDOCK modeling were defined as those involved in the Hsp90-p23 interactions according to [38]. Passive residues were defined as residues within a 5A radius of active residues. (DOCX) [file pone.0071936.s003.docx]

| **Interaction** | **AIR Template** | **Hsp90 Residue Number** | **Chain ID** | **P23 Residue Number** |
| --- | --- | --- | --- | --- |
| Interaction 1 | Active Residues | 12-21,151-155 | A | 31-37,85-91 |
| Interaction 2 | Active Residues | 27,387 | A | 121,122,124 |
| Interaction 3 | Active Residues | 315,375,388,391 | A | 19-22,118-120,122 |
|  | Active Residues |  |  | 123,125,126 |
| Interaction 4 | Active Residues | 94-125 | B | 13-16,113-118 |
