# Supplementary material for: Differential Modulation of Functional Dynamics and Allosteric Interactions in the Hsp90-Cochaperone Complexes with p23 and Aha1: A Computational Study
Source: PLoS One. 2013 Aug 19;8(8):e71936. doi: 10.1371/journal.pone.0071936 (PMC3747073; doi:10.1371/journal.pone.0071936)
Supplement: Table S2 — The AIR Template for HADDOCK Simulations of the Hsp90-Aha1 Complex. The AIR sets used in HADDOCK to generate the predicted docked complexes defined the active residues only by the crystal structure of the Aha1-N bound with the yeast Hsp90-M domain [66]. Passive residues were defined as residues within a 5A radius of active residues. The selected panel of active residues included primarily the hydrophobic Hsp90-M residues L315, I388, V391 and the Aha1-N residues I64, L66, F100. The passive residues were defined as residues within 5Å of the actives. (DOCX) [file pone.0071936.s004.docx]

| **Interaction** | **AIR Template** | **Hsp90 Residue Number** | **Aha1 Residue Number** |
| --- | --- | --- | --- |
| Interaction 1 | Active Residues | 315, 388, 391 | 64, 66, 100 |
|  | Passive Residues | 311,312,313,314,316,317 | 53,55,56,57,58,59 |
|  | Passive Residues | 371,373,375,383,385,386 | 62,63,65,67,68,98 |
|  | Passive Residues | 387,389,390,392,393,394 | 99,101,102,143,147 |
|  | Passive Residues | 395,396 |  |
| Interaction 2 | Active Residues | 314 | 64,65 |
|  | Passive Residues | 312,313,315,316 | 54,55,56,57,58,59 |
|  | Passive Residues |  | 62,63,66,67,100 |
| Interaction 3 | Active Residues | 387, 390, 394, 398 | 53, 68, 97, 101 |
|  | Passive Residues | 356,385,386,388,389,391 | 54,55,56,57,58,59 |
|  | Passive Residues | 392,393,395,396,397,399 | 62,63,66,67,100,146 |
|  |  | 400,401,402,403 | 147 |
| Interaction 4 | Active Residues | 434 | 108 |
|  | Passive Residues | 432,433,435,436,437,438 | 94,95,96,97,105,106 |
|  | Passive Residues |  | 107,109,110,136 |
| Interaction 5 | Active Residues | 433 | 96 |
|  | Passive Residues | 428,429,431,432,434,435 | 68,69,70,71,94,95 |
|  | Passive Residues | 436,437,439 | 97,98,108,109,110 |
| Interaction 6 | Active Residues | 469 | 122 |
|  | Passive Residues | 464,466,467,468,470,471 | 40,79,119,120,121,123 |
|  |  | 518,519,520 | 124,125,126,127 |
| Interaction 7 | Active Residues | 514 | 110 |
|  | Passive Residues | 436,510,511,512,513,515 | 92,93,94,95,96,108 |
|  |  | 516,519,520,521,583 | 109,111,112 |
| Interaction 8 | Active Residues | 515 | 128 |
|  | Passive Residues | 436,512,513,514,516,517 | 111,123,124,125,126,127 |
|  |  | 518,519,520,521 | 129,130,131,132,133,136 |
| Interaction 9 | Active Residues | 300 | 19,23 |
|  | Passive Residues | 293,294,298,299,301,302 | 15,16,17,18,20,21, |
|  |  | 326 | 22,24,25,26,27,28, |
|  |  |  | 138,139,141,142,145 |
|  |  |  | 146,149,150 |
